# Computational approaches in chemical space exploration for carbon fixation pathways

**DOI:** 10.1038/s41540-025-00641-8

**Published:** 2026-01-08

**Authors:** Anne-Susann Abel, Nino Lauber, Jakob Lykke Andersen, Rolf Fagerberg, Daniel Merkle, Christoph Flamm

**Affiliations:** 1 Institute for Theoretical Chemistry, University of Vienna, Vienna, Austria; 2 Department of Mathematics and Computer Science, University of Southern Denmark, Odense, Denmark; 3 Algorithmic Cheminformatics Group, Faculty of Technology & Center for Biotechnology (CeBiTec), Bielefeld University, Bielefeld, Germany

**Keywords:** Biochemistry, Chemistry, Computational biology and bioinformatics, Mathematics and computing

## Abstract

Chemical space exploration is an important part of chemistry and biology, enabling the discovery and optimization of metabolic pathways, advancing synthetic metabolic functions, and understanding biochemical network evolution. We use a graph-based computational approach implemented in the cheminformatics software MØD, integrated with Integer Linear Programming (ILP) optimization, to systematically search chemical spaces. This approach allows for flexible and targeted queries, including identification of autocatalytic cycles, thermodynamic considerations, and discovery of novel enzymatic cascades. Specifically, we explore the chemical space of natural and artificial carbon fixation pathways defined from relevant enzyme reactions. By applying different optimization criteria, we identify new varieties and recombinations of natural autocatalytic pathways, and compare the properties of the pathways. This work highlights the versatility of graph-based cheminformatics for the purpose of chemical space exploration and artificial pathway design. Potential applications of this framework extend to carbon capture technologies, improved agricultural yields, and value-added chemical production, advancing efforts to address global sustainability challenges.

## Introduction

As we fight climate change, reducing greenhouse gas emissions to a net zero is crucial. One strategy for this is the removal of CO_2_ from the atmosphere, ideally turning it into value added chemicals for further use^1,2^. At its core, the process of carbon fixation in living organisms reduces carbon from its most oxidized form (CO_2_ or ${{\rm{HCO}}}_{3}^{-}$) to valuable metabolic building blocks, e.g. sugars. This is a thermodynamically unfavorable reaction, making large-scale implementation a challenge^1^.However, nature has evolved solutions using enzymatic catalysis and biochemical pathways, coupling more unfavorable reactions to more favorable ones^3,4^. To date, seven natural carbon fixation pathways have been identified, with several additional artificial pathways proposed^5–13^.

The Acetyl-CoA-Succinyl-CoA pathway family^3^ is a particularly interesting group, where each of the pathways overlaps significantly with at least one other structurally. Additionally, they all exhibit autocatalytic activity. Both of these properties make them highly relevant as templates for designing artificial pathways. These pathways and their autocatalytic activity are described in more detail in the Methods section.

Autocatalysis is at the heart of many pathways in the central carbon metabolism^14^, and therefore an interesting property to look for when combining reactions of the carbon metabolism from different organisms to design novel pathways, as demonstrated by ref. ^15^. Previous advances in artificial pathway design, such as the work of the Erb Lab^16,17^, demonstrate that combining reactions from diverse domains of life and optimizing them for thermodynamic favorability can yield novel, highly efficient pathways. These previous approaches^15–17^ use a combination of heuristic considerations and conceptual analysis, thermodynamic optimization, database searches, and an extensive experimental phase with several steps of optimization, including site-directed mutagenesis to enhance the kinetic properties of the involved enzymes. Hence, they constitute highly time intensive, manual efforts with the goal of subsequent implementation of specific pathways in an in vitro setting, while computational tools play a minor role in the design and optimization process.

However, such recombinations of pathways can be further explored by computational approaches. Previous work on computational exploration of the carbon fixation space^18^ focuses on an in-depth analysis of available data from databases with manual curation, flux balance analysis, and activity analysis for known artificial and natural pathways. The proposed modified pathways are then manually curated following expert intuition^18^. This approach requires complete knowledge of the parameters for every involved enzyme, making generative experiments infeasible.

In this paper, we focus on the computational angle of pathway design itself, for which we propose an approach based on generative chemical space expansion, pathway queries, and topological optimization of pathway solutions based on thermodynamic annotation. The overall goal is fast and flexible pathway suggestions for speeding up the design process.

**Fig. 1:**
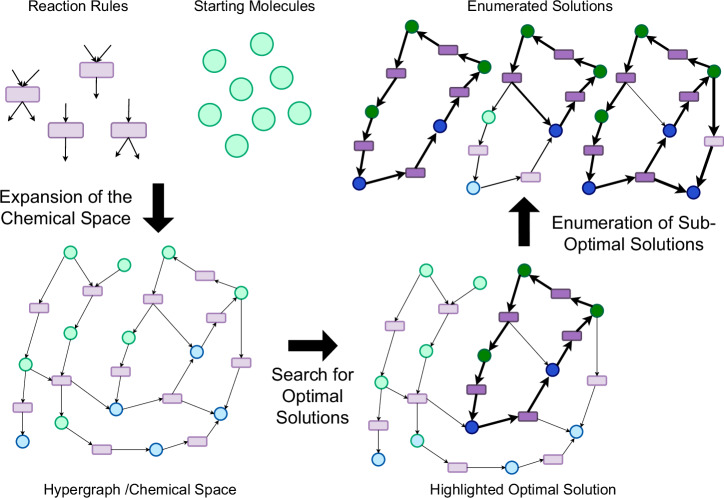
Schematic graphical abstract of the approach for pathway design applied in this study. Graph-grammatical building blocks like rules (in purple) and starting molecules (in green) are used to expand a chemical space. During the expansion, new molecules are created (in blue). On this space, an ILP search is performed to find pathways, where the solutions are evaluated. Highlighted in darker colors are the different pathway solutions.

In detail, we present a graph-based computational approach using the cheminformatics software MØD^19^, which includes facilities for pathway search via Integer Linear Programming (ILP) optimization^20^. This method allows us to systematically construct and explore chemical spaces with high flexibility, enabling targeted queries for user defined questions, like finding autocatalytic cycles and searching for alternative products. A graphical overview of the approach is presented in Fig. 1.

Our approach starts by iteratively expanding a chemical space, specified by a set of reaction rules and a pool of initial molecules, into a chemical reaction network (CRN). The CRN is constructed as a directed hypergraph, wherein the hyperedges are the reactions and the vertices are the molecules. Reactions are described as reaction rules, each rule representing a general molecular transformation—i.e., a reaction class—instead of a specific reaction (see Fig. 5 for an example). This method of reaction formalization as rules is a common approach for systems biology^21,22^. It allows the model to capture enzyme promiscuity and account for unconventional or novel metabolic transformations. A detailed list of the transformation rules used in this work can be found in Figure S1.

The initial pool of molecules used to start the stepwise expansion of the CRN consists of known metabolites and common cofactors typically present in the organisms of interest. As the reaction rules are applied to the input set of molecules, they generate new molecules and reactions, progressively expanding the chemical space. Novel molecules will form through the application of promiscuous reaction rules on the input molecules, achieving the expanded chemical space of carbon fixation, represented as a hypergraph, after a set number of expansion steps. A detailed list of the initial molecules used in this work can be found in Tables S1 and S2.

On this directed hypergraph representing the expanded chemical space, the search for pathways is achieved through hyperflow queries^20^, which are described in detail in the Methods section. In short, a hyperflow is a route through a hypergraph, in this case through a CRN, with an inflow and outflow of some molecules. The net reaction represented by the hyperflow is the flow on the input and the output molecules. The flow on molecules throughout the rest of the route balances out, i.e., each molecule is produced and consumed in equal amounts. Thus, a hyperflow corresponds well with the standard notion of a pathway. A hyperflow query is the search for such a route with a specified structure. The search query can for instance require certain input and output molecules as start and/or end points, as well as forbidden or preferred reactions or elements of the route. These specifications are fed into an ILP model, which is a set of linear equations, with an objective function to be solved and constraints to be satisfied that combined model the desired hyperflow structure. In our work, we use the hyperflow queries to find the shortest carbon fixation pathways in the generated CRN. Specifically, our queries ask for flows minimizing both the overall number of hyperedges (reactions) used and the absolute flow on these respective edges. Since we are interested in autocatalytic pathways, the only allowed inflow and outflow for the model is the autocatalyst itself and cofactors, as well as CO_2_ or ${{\rm{HCO}}}_{3}^{-}$. This way, the net reaction for a solution to the query represents the production of one more autocatalytic molecule under the usage of cofactors and the fixation of one or more carbon units.

The feasibility of the solutions found is evaluated by a post-annotation workflow in three ways. First, the length of a pathway solution is compared to pathways from the literature. Second, the number of cofactors is counted as a measure for energy units and electrons used in the pathway. Third, the ${\Delta }_{r}{G}^{{\prime} \circ }$ of the reaction is calculated as a measure for the thermodynamic feasibility. This reaction energy ${\Delta }_{r}{G}^{{\prime} \circ }$ is calculated by subtracting the combined energies of formation of the educts from the products, with the energies of formation being obtained from using the eQuilibrator computational framework^23,24^. The workflow is summarized in Fig. 1 and a detailed description of the workflow can be found in the Methods section. It is worth noting that although we illustrate our workflow as an approach for carbon fixation pathway design, applying it to the chemical space of natural and artificial carbon fixation, the generality of the methodology allows it to be used for pathway investigations on any chemical space of interest.

## Results

The key findings of this work can be summarized as follows: We suggest novel synthetic carbon fixation pathways, found through recombining elements of the biochemical pathways of the carbon fixation space, that have qualities similar to those of the natural pathways and of some of the most effective synthetic pathways. These key findings are represented in Table 1. In addition, we demonstrate the computational efficiency of our methodology by providing 1000 solutions to a variety of pathway queries.

**Table 1:** A comparison of the flow solutions as proposed theoretical carbon fixation pathways with selected theoretical, synthetic, and natural pathways, focusing on shortest pathways specifically

Pathway	Status	Steps	ATP units	Co-factors	Carbon units	ATP/ Carbon unit	Co-factors/ Carbon unit	Ref.
Shortest autocatalytic cycle, Acetyl-CoA	Theoretical	11	2	5	2	1	2.5	this work
Shortest autocatalytic cycle, Malate	Theoretical	12	3	8	4	0.75	2	this work
CETCH	Synthetic	11	1	4	2	0.5	2	^16^
rGPS-MCG	Synthetic	18	4	6	3	1.33	2	^15^
C4-glyoxylate / alanine option	Theoretical	9	2	2	2	1	1	^26^
rTCA	Natural	12	4	7	4	1	1.75	^5,26^
3HP/4HB	Natural	16	4	6	2	2	3	^8,26^
DC/4HB	Natural	14	4	7	2	2	3.5	^9,26^
3HP-bicycle	Natural	19	3	4	2	1.5	2	^10,26^

Pathway: name of the autocatalytic carbon fixation cycle. Status: implementation status of a given pathway, divided into the categories theoretical, implemented as synthetic, or known natural pathway. Steps: number of steps necessary to complete one autocatalytic cycle and produce one additional molecule of the autocatalyst. ATP units, Cofactors, Carbon units: number of units or molecules needed for one autocatalytic cycle to complete. ATP / Carbon unit, Cofactors / Carbon unit: ATP and cofactors normalized against carbon units fixed during one cycle. *Ref.* Reference paper where the pathway was discovered or described, *CoA* Coenzyme A, *CETCH* Crotonyl-CoA/ethylmalonyl-CoA/hydroxybutyryl-CoA, *rGPS-MCG* reductive Glyoxylate Pyruvate synthesis - Malyl-CoA-glycerate, *rTCA* reductive tricarboxylic acid, *3HP/4HB* 3-hydroxypropionate4-hydroxybutyrate, *DC/4HB* dicarboxylate.

## New Pathways and Comparison to Literature

Various comparison studies of autocatalytic carbon fixation cycles have introduced the concept of comparing a set of key measures^4,16,25^ to understand the classification of a novel pathway. These measures include the number of steps required to complete one cycle, the ATP units and cofactors used in the completion of this cycle, and the carbon units fixed per cycle.

In this work, we propose two novel theoretical pathways found via our methodology. The two presented pathways are the result of flow queries searching in the CRN for the shortest pathway (that is, with the fewest number of reactions) adhering to the structural definition of autocatalytic cycles in the Methods section.

The CRN was build using rules derived from the reactions in the autocatalytic carbon fixation cycles of the Acetyl-CoA-Succinyl-CoA pathway family^3^ and in a selection of artificial cycles^16,26^. The information for the enzymatic reactions involved was taken from the KEGG database^27^. A detailed description of the chemical space composition can be found in the Methods section.

The autocatalyst, i.e., the inflow and outflow molecule, in the two searches were Acetyl-CoA and Malate, respectively, and the query specified the production of one additional molecule of the autocatalyst while fixing carbon in the form of CO_2_ or ${{\rm{HCO}}}_{3}^{-}$. This search was performed on the CRN obtained after two expansion steps and characterized in the second line of Table 4. The two proposed theoretical pathways we refer to according to their search objectives as Shortest autocatalytic cycle Acetyl-CoA, and Shortest autocatalytic cycle Malate. In Table 1, we compare their characteristics to benchmark pathways from other studies. The explicit structure can be found in the GitHub repository under Output Pathways https://github.com/anne-susann/C_fixation_pathway_design.

These benchmark pathways are either natural, theoretical, or synthetic, the last phrase meaning that they have been theoretically designed and then implemented in vitro. The Acetyl-CoA pathway requires 11 steps to generate one autocatalytic molecule, and the Malate pathway takes 12 steps. Compared to the natural pathways, the two novel suggestions either use strictly fewer or the same number of steps as the shortest natural one. Compared to the synthetic pathways, the novel suggestions are in the range of the shortest synthetic pathway in terms of the number of steps used. Especially when comparing the cofactor and ATP usage normalized against the number of carbon units fixed between pathways, the efficiency of the newly proposed theoretical pathways is close to or better than the most efficient pathways. ATP units and cofactors as energy and reduction equivalents give a cost measure for cells or in vitro systems. The Acetyl-CoA pathway requires one unit more of ATP (2 in total) and one more reduction-oxidation (redox) cofactor (5 in total) than the highly efficient CETCH cycle while fixing the same amount of carbon units per cycle, namely two. It is comparable in ATP and cofactor usage to natural carbon fixation cycles when normalizing for carbon units. The Malate pathway has a higher total requirement, with 3 ATP units and 8 redox cofactors, but fixing 4 carbon units in the process, making it more efficient than the Acetyl-CoA cycle in both ATP and cofactor use. The Malate cycle is also only 0.25 cofactors per carbon unit away from the shortest natural pathway, rTCA, with respect to these cofactor requirements.

## Exploration Scope of the Method

**Fig. 2:**
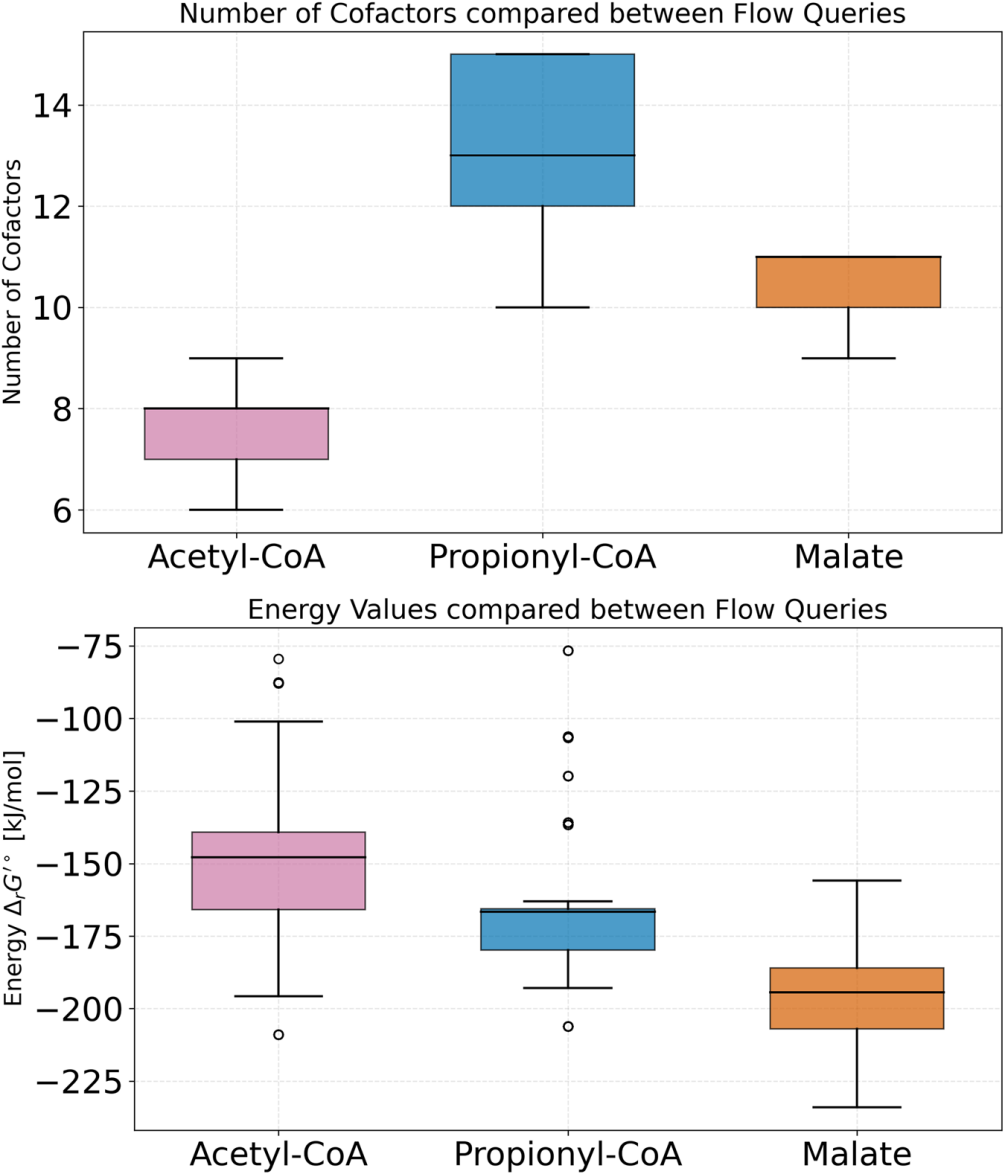
Comparison of cofactor usage and reaction energy ${\Delta }_{r}{G}^{{\prime} \circ }$ for 1000 solutions to different flow queries. The cofactors include ATP units and redox cofactors such as NAD(P)H and Ferredoxin. The three queries are looking for the shortest autocatalytic cycle toproduce Acetyl-CoA, Propionyl-CoA, and Malate, respectively.

The exploratory potential of our approach includes the search for many good solutions, not just a single best. Three different queries for autocatalysis, using Acetyl-CoA, Malate, and Propionyl-CoA as autocatalytic molecules respectively, were taken as benchmark searches for investigating the differences in the solutions for different biological products. The flow query was built to find 1000 solutions satisfying the query constraints. Within those, as long as the objective value (that is, pathway length) is the same, the solutions are considered equally good by the ILP solver. However, each of the 1000 solutions was required to be topologically different. Additionally to the pathway queries described in Table 1, the Propionyl-CoA query is included here. Whereas the previous section focused on the shortest pathways, especially in comparison to other known pathways, the following highlights more the exploratory potential, allowing for solutions that are longer to be considered as well. These 1000 solutions were then evaluated statistically by the number of cofactors that are used in each solution and by the reaction energy ${\Delta }_{r}{G}^{{\prime} \circ }$ value of those solutions, visualized in Fig. 2.

The two measures shed light on two different parameters for a potential implementation, namely cofactor requirement and thermodynamic feasibility. A more negative ${\Delta }_{r}{G}^{{\prime} \circ }$ makes a pathway in general more likely to react in the forward direction, while the cofactor requirements are generally considered the standard measure of energy efficiency in systems biology. While a negative value for ${\Delta }_{r}{G}^{{\prime} \circ }$ is required, the amount of cofactors used in a pathway heavily influences its implementation potential, since cofactor-hungry pathways require more input of expensive cofactors and the addition of recycling pathways to the setup.

The results show that the Acetyl-CoA pathway has the lowest usage of cofactors, with an average of 7.6 cofactors used (see Fig. 2 and Table 2). The Propionyl-CoA and Malate pathways need more cofactors, with an average of 13.3 and 10.6. Since the latter two pathways are longer in steps, the cofactors were also calculated per step of the pathway. This results in a cofactor-per-step ratio of 0.69 for Acetyl-CoA, and 0.89 and 0.88 for Propionyl-CoA and Malate (see Table 2), making the autocatalytic Acetyl-CoA cycle not only the shortest but also the most cofactor-efficient pathway.

**Table 2:** Flow query results for the 1000 solutions to find the shortest autocatalytic carbon fixation pathway, for Acetyl-CoA, Malate, and Propionyl-CoA, respectively

Autocat. molecule	Steps average	Co-factors average	Co-factors per step	Energy average [kJ mol^−1^]
Acetyl-CoA	11	7.6	0.69	−150.66
Propionyl-CoA	15	13.3	0.89	−165.82
Malate	12	10.6	0.88	−196.98

The table shows the autocatalytic molecule that was searched for, the average length of the pathway solution as steps, the average cofactor use and the cofactor use normalized against the pathway length.

The energy measure shows Malate having the lowest energy value for the pathway, with ${\Delta }_{r}{G}^{{\prime} \circ }=-196\,{\rm{kJ}}\,{{\rm{mol}}}^{-1}$ on average. The Acetyl-CoA pathway has the highest energy with ${\Delta }_{r}{G}^{{\prime} \circ }=-150\,{\rm{kJ}}\,{{\rm{mol}}}^{-1}$, and Propionyl-CoA is in the middle with ${\Delta }_{r}{G}^{{\prime} \circ }=-165\,{\rm{kJ}}\,{{\rm{mol}}}^{-1}$. In this measure, the Malate pathway seems to be the most thermodynamically driven, especially when regarding the need for one more step compared to Acetyl-CoA but still having a lower energy value. However, these ${\Delta }_{r}{G}^{{\prime} \circ }$ values include the energy gained from the used cofactors.

## New Acetyl-CoA Producing Pathways

**Fig. 3:**
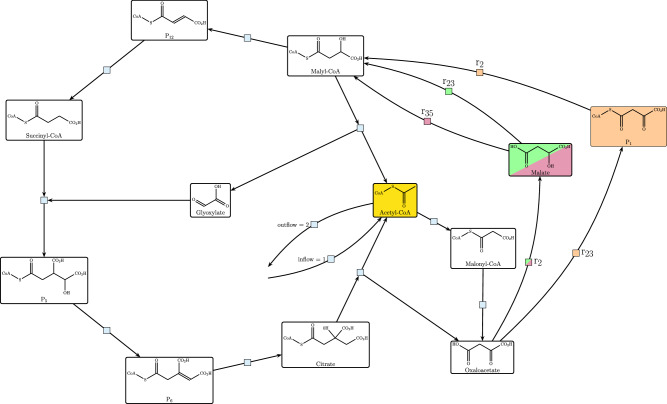
Comparison of the first 3 solutions for finding the shortest autocatalytic cycle producing Acetyl-CoA. The important differences are in the rules used in the solution and the molecules used. Reaction rules used are modeled after the following enzymatic reactions: *r*_16_ Acetyl-CoA-carboxylase (3HP4HB), *r*_9_ 2-ketoglutarate:ferredoxin oxidoreductase (rTCA), *r*_2_ ATP citrate lyase (rTCA), *r*_23_ 4HB-CoA synthetase (3HP4HB), *r*_35_ succinyl-CoA malate CoA transferase (3HP bicycle), *r*_20_ 3HP-CoA dehydratase (3HP4HB), *r*_5_ fumarate reductase (rTCA), *r*_54_; *r*_6_ fumarase (rTCA), *r*_7_ aconitase (rTCA), *r*_8_ crotonase (rTCA), *r*_1_ succinyl-CoA synthetase (rTCA).

As a benchmarking example, three individual solutions from the solution space for shortest autocatalytic Acetyl-CoA production were further inspected. The solutions were chosen based on heuristic considerations on topological comparability from the solution space mentioned before. The topological structure of the three solutions is very similar, hence we can overlay and compare their specific differences in steps. In Fig. 3, the common core of the three solutions is the parts where molecules are shown on a white background while the respective differences are marked by green, pink, and orange. Acetyl-CoA as the autocatalytic molecule, marked in yellow, is at the center of the pathways. Much of the pathway relies on the rTCA as the basic structure, but we find a glyoxylate shunt in between, as well as a combination of other reactions of the Acetyl-CoA-Succinyl-CoA space. An important branch point between the three solutions is on the route between Oxalacetate and Malyl-CoA, where we observe different combinations of enzymes used and intermediate molecules produced.

Because of the difference in enzymes used between the compared solutions, the cofactor usage also changes. In Table 3, the inflow and outflow of cofactors for each solution is detailed. The main difference between the pathways is the ATP usage, with solution 1 (Sol 1 in Table 3) having the lowest ATP consumption of only one. This yielded a higher ${\Delta }_{r}{G}_{total}^{{\prime} \circ }$ value, but with −80 kJ mol^−^^1^ the pathway is still thermodynamically feasible and has a low ATP usage.

**Table 3:** Detailed description of the inflow and outflow of molecules as well as the overall reaction energy for the three flow solutions depicted in Fig. 3

		Sol 0	Sol 1	Sol 2
Inflow molecules	Acetyl-CoA	1	1	1
	CoA-SH	1	1	1
	ATP	1	1	3
	ADP	2	0	0
	NADH	1	1	1
	NADPH	2	2	2
	Ferredoxin (red)	2	2	2
	H^+^	6	6	6
	CO_2_	1	1	1
	${{\rm{HCO}}}_{3}^{-}$	1	1	1
Outflow molecules	Acetyl-CoA	2	2	2
	ADP	1	1	1
	AMP	2	0	2
	NAD^+^	1	1	1
	NADP^+^	2	2	2
	Ferredoxin (ox)	2	2	2
	P_*i*_	3	1	1
	PP_*i*_	0	0	2
	H_2_*O*	1	3	1
	${\Delta }_{r}{G}_{total}^{{\prime} \circ }$ [kJ mol^−1^]	−138	−80	−168

The flow solutions were optimized to find the shortest autocatalytic pathway to produce Acetyl-CoA in the carbon fixation space.

A closer view on the three flow solutions shows the combination of natural pathways to form novel pathways, as on the topological level, we find a reordering of enzymatic reactions and recombination of reaction rules with different natural pathway origins.

## Chemical Space Design

To expand the chemical space of carbon fixation into a CRN, input molecules were defined and reaction rules were applied to those molecules. The input molecules consist of 49 general molecules that are intermediates of the Acetyl-CoA-Succinyl-CoA pathways^4^, the synthetic CETCH cycle by Schwander et al.^16^, and the theoretical glyoxylate cycle proposed by Bar-Even et al.^26^, and 20 helper molecules, which include molecules like various cofactors, water, and CO_2_. A detailed listing of the input molecules can be found in Tables S1 and S2.

The smallest possible CRN in our setup contains the input rules of the carbon fixation cycles as described above.This space was created via one expansion step, leading to 165 vertices, corresponding to molecules and 220 hyperedges, corresponding to reactions, as described in Table 4.

**Table 4:** Characterization of the CRN for Carbon Fixation after different expansion rounds

Expansion Steps	# Vertices (Molecules)	# Hyperedges (Reactions)
1	165	220
2	318	942
5	996	29266

The number of expansion steps represents how often the rules were applied on the chemical space, and the numbers of vertices (molecules) and hyperedges (reactions) describe the size of the CRN.

Performing flow queries, i.e., the search for an autocatalytic carbon fixation pathway through the reaction network as described in the Introduction and in full detail in the Methods section, found only the pathways given as input. No recombinations (i.e., no cross connections between the natural and/or synthetic and theoretical pathways) within the chemical space took place with only one expansion step.

With two expansion steps, the number of reactions increases to 942, while the number of molecules nearly doubles to 318 (see Table 4). This is the space that subsequent analysis were performed on. This CRN proved big enough to have novel compounds and novel cross connections and pathways, but does not reach a size where running flow queries requires a large amount of computation time.

We experimented with further expansion steps up to and including five, at which point combinatorial growth rendered flow queries computationally infeasible. While larger expansions were technically possible, they resulted in disproportionately large networks with limited additional insight. Earlier steps (three and four expansion steps) were explored, but the two-step expansion space already supported sufficient pathway choices and kept computation times for flow queries down.

## Discussion

In this paper, we present a new method to explore the chemical space of carbon fixation for novel pathway combinations as well as artificial pathways, and we give examples of novel pathways. In our experiments, the space was restricted to be based on known natural, synthetic, and theoretical carbon fixation pathways, but the method can be generalized to any chemical space, natural or not, involving any number and types of reactions. The chemical space expansion strategy allows for a flexible design of the CRN and an exploratory approach to investigate the chemical space.

We found two interesting novel pathways after applying our search framework, one producing Acetyl-CoA and the other producing Malate. Both autocatalytic cycles have quality measures comparable to other artificially designed pathways and the most efficient natural pathways, as detailed in the Results section.

The lower energy value of the Malate pathway makes it an interesting contender for potential implementation. However, we note that the overall cofactor usage of the Acetyl-CoA pathway as described in Fig. 2 and Table 2 is lower while the pathway still has a prominent negative ${\Delta }_{r}{G}^{{\prime} \circ }$ as a driving force.In a wet-lab implementation setting, this means that less cofactors need to be supplied and recycled by a cofactor recycling system. Since cofactor availability can pose a significant challenge, it likely makes the Acetyl-CoA pathways the more interesting platform for further exploration.

Another factor to be considered with respect to feasibility of implementation is the specific cofactors used in the solutions. The pyruvate synthase (pyruvate:ferredoxin oxidoreductase) and the ketoglutarate synthase (2-ketoglutarate:ferredoxin oxidoreductase) are both highly efficient carboxylating enzymes found in the rTCA.They use Ferredoxin as the reduction cofactor, which has a higher reduction potential than NAD(P)^3^. We can see in Table 3 that the solutions compared here all apply Ferredoxin, as do the solutions explored in the comparison of the 1000 solutions. However, Ferredoxin is an oxygen-sensitive cofactor, meaning implementation would have to be under anaerobic conditions. This could be a major hurdle for the in vitro implementation of the suggested pathways, but it could possibly be achieved through metabolic engineering of anaerobic strains^28^.

Further considerations for implementation include enzyme kinetics. Even though the thermodynamic potential indicates a driving force for the pathways, enzyme kinetics could lead to non-favorable conditions. A slow reaction could cause a bottleneck for the pathway, leading to (intermediate) product build up and potential inhibitory effects^29^. These kinetic considerations have been solved in the past for other projects, with solutions including kinetic modeling (for known kinetic constants), enzyme engineering, and directed evolution^16,30,31^.

A deeper look into the composition of the Acetyl-CoA pathway solutions revealed that the combination of enzymes from different natural origins yields shorter and more efficient pathways. The specific combination in each solution can differ. At the same time, the length of the pathway stays the same, while the cofactor usage differs. This shows the versatility of the overall chemical space and highlights the potential to get many pathway suggestions, which can then be filtered to satisfy relevant criteria for later implementation, such as oxygen-sensitivity, ATP usage, or redox cofactor usage.

The challenge arising with enzymes from different biological origins could be enzyme availability. Some organisms have been more extensively studied, and their enzymes are therefore more widely available and easier to handle in a lab context. As with previous implementation challenges, enzyme engineering and directed evolution could be potential solutions for these issues^29^.

While these described challenges to wet-lab implementation are a concern to keep in mind, the value in this work still lies within the design aspect. Idea generation and pathway design are integral parts to a subsequently successful implementation in a lab context. Successful in vitro synthetic pathways stem from previous modeling by the groups themselves^16,17^, or are direct implementations^32^ of suggested theoretical pathways^20^.

We were able to achieve our results of theoretical pathway design while using a strictly topological approach for pathway searches, with ILP as the search algorithm for novel pathways. This approach, augmented with a post-annotation workflow for feasibility measures, gives a flexible and robust approach for exploratory pathway searches in any chemical space. At the same time, there is a minimal need for extensive database searches and the associated computational cost is low, as all of the workflow can be performed on a standard laptop.

The advantage of our approach is the possibility to individualize the optimization criteria, to include as many constraints as wanted, and to explore beyond the scope of what is known already. By the rule based expansion of the CRN, it can produce molecules previously unknown to a specified chemical space and can apply the given reactions to those molecules as well, while still adhering to the chemical principles of a reaction network^33^. Kinetic modeling for these pathways using ordinary differential equations is possible with this approach as well, but it was not the focus of this study. Future work should consider kinetic measures as well as focus on including cofactor recycling systems, in order to make the proposed solution less cofactor expensive and more promising to implement in a wet lab setting. Future design efforts may also focus on incorporating metabolic reactions not directly involved in carbon fixation pathways to look for promising short cuts.

## Methods

**Fig. 4:**
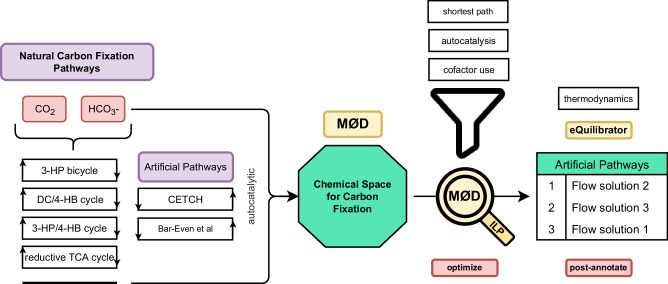
Workflow for using a graph-based rule-based approach for the exploration of the chemical space of natural and artificial carbon fixation. First, the natural and artificial pathways are turned into transformation rules and the chemical space is expanded. The expansion is restricted to molecules with up to 6 carbon atoms and molecules with no more than one CoA attached. Then, flow queries are used to search and optimize for a given objective function with constraints, yielding a list of flow solutions. The constraints include only allowing for cofactors to be inflow and outflow molecules, and a net production of one autocatalytic molecule. Lastly, post-processing allows for ranking of solutions. 3-HP 3-hydroxypropionate, DC dicarboxylate, 4-HB 4-hydroxybutyrate, TCA tricarboxylic acid cycle, CETCH crotonyl-CoA/ethylmalonyl-CoA/hydroxybutyryl-CoA cycle^16^, Bar-Even et al. proposed pathways from Bar-Even and his colleagues^26^.

The exploration of the carbon fixation space and the search for artificial pathways followed the workflow shown in Fig. 4.The workflow can be split into the following tasks: (i) selection of the chemical space that will be modeled, (ii) expansion of the chemical space using MØD, (iii) optimization and pathway search, and (iv) post annotation of pathway solutions. Each of these tasks will be explained in detail in the following.

## Modeling the Chemical Space of Carbon Fixation

The chemical space of carbon fixation was formalized using MØD^19^ (version 1.0.0), a graph- and rule-based cheminformatics tool capable of constructing chemical reaction networks as directed hypergraphs. A detailed description of the software MØD, including the hypergraph construction, can be found in Andersen et al.^19,34,35^.

The hypergraph in our approach is the representation of the CRN. A CRN generally consists of a set of molecules and a set of reactions. This can be modeled as a directed multi-hypergraph *H* = (*V*, *E*), with the set of vertices *V* representing the molecules, and the set of directed hyperedges *E* representing the reactions. Each hyperedge *e* ∈ *E* contains a pair (*e*_*tail*_, *e*_*head*_) of multisets of vertices *e*_*tail*_ ⊆ *V* and *e*_*head*_ ⊆ *V*, corresponding to the molecules that flow in and out of a reaction. Hyperedges, as opposed to simple edges, allow the direct modeling of many-to-many relations between reactant and product molecules.

**Fig. 5:**
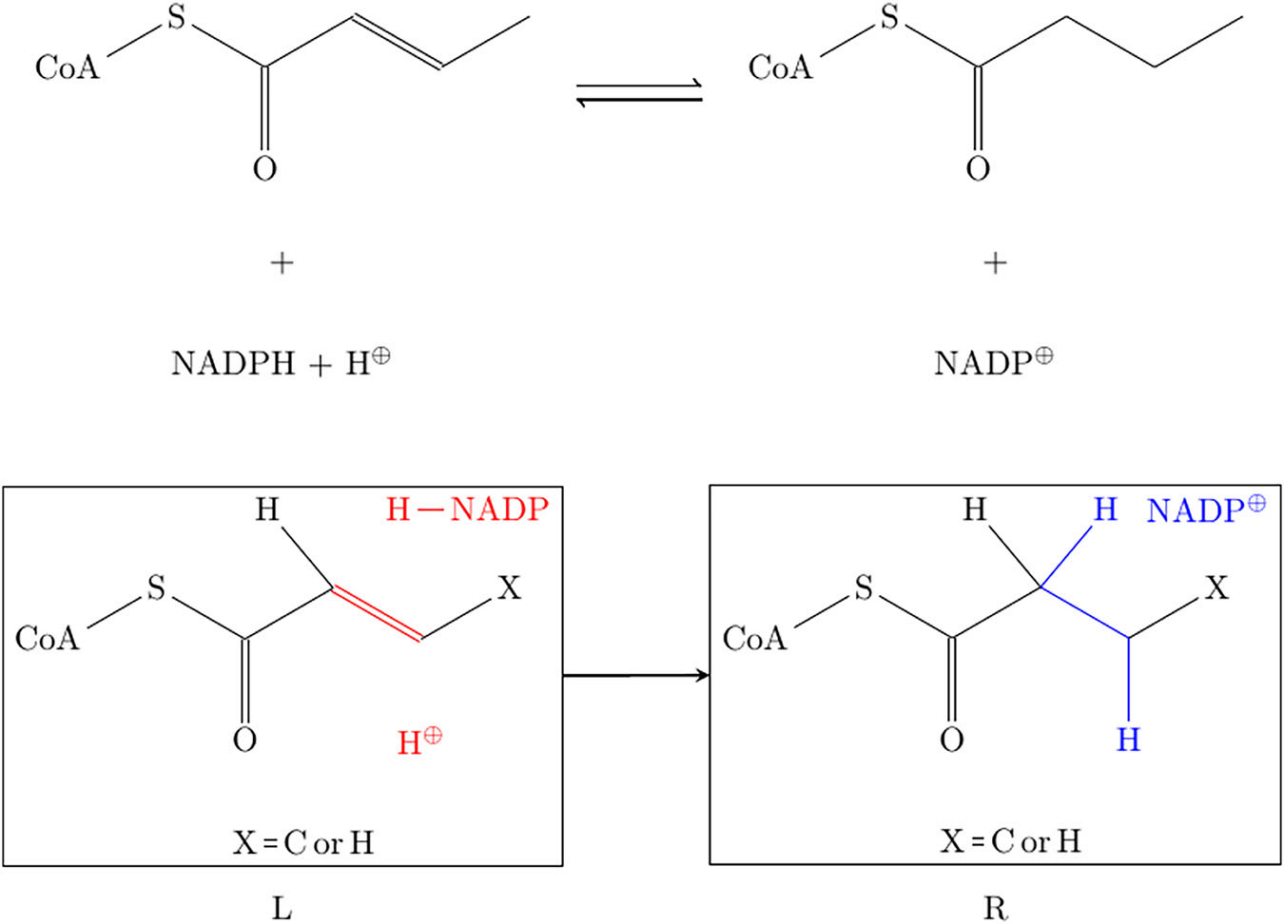
Example of a transformation rule as used by MØD. This specific rule shows the acrylyl-CoA reductase as found in the 3HP/4HB cycle of carbon fixation. The mechanism was modeled after the respective KEGG entry for this enzyme^27^. The top shows a traditional representation of the enzymatic reaction, while the bottom shows the representation as a graph transformation rule, with the left (L) and the right (R) side as the bonds before and after the reaction. X is a variable that can define different atoms, in this case C or H.

CRNs in MØD are expanded using three core elements. Firstly, rules represent the (bio)chemical reactions and define how molecules are transformed. Rules operate as graph transformations that rewrite molecular graphs, breaking or forming bonds and adjusting charges. Depending on the molecular context attached to the reaction center of the rewrite rule, the specificity of the rewrite rule may be tuned. In this way one rule may represent multiple reactions that induce the same net change in the molecules it may be applied to. For example, a generic reduction rule with no context around the reaction center will perform all reactions catalyzed by oxidoreductases like reductases or dehydrogenases (see Fig. 5). This allows for a flexible construction of the chemical space, not needing to formalize every single reaction but working with reaction types and classes.

The rules in this work are formalized in the Graph Modeling Language (GML) format^36^. This format allows us to follow consistent atom-atom maps, perform reliable subgraph matching, and gives full control over the context of the reaction.

Secondly, molecules serve as the input graphs upon which the rules are applied, expanding the network from a set of core molecules. The initial molecules for the network expansion are listed in Table S1 and S2. Finally, expansion strategies define how the rules are applied iteratively to the given molecules to construct the reaction network step-by-step. A more detailed description of CRN expansion in MØD can be found here^34^. For the networks built here we used an expansion strategy that restricts the chemical space to molecules with at most 6 C atoms, to avoid a combinatorial explosion and stay close to nature in this approach. All of the included natural pathways and the known artificial pathways have no molecules involved that go beyond 6 C atoms in the backbone of the molecule. The carbon atoms in the covalently bound cofactor Coenzyme A (CoA) are not considered for this 6 C restriction. Further, the expansion was restricted to produce molecules with at most one CoA attached to them, to maintain biochemical validity and stability in the CRN.

The rules were derived from enzymatic reactions available in the KEGG database^27^. For this study, the molecular context for the reactions were designed conservatively to reflect enzymatic specificity while allowing for some flexibility that could reasonably be reached with enzyme isoforms, enzyme engineering or enzyme promiscuity. The detailed rule formulation can be found in Figure S1, where the context of each rule is depicted. This study focused on autocatalytic carbon fixation cycles, excluding pathways like the Wood-Ljungdahl pathway and the reductive glycine pathway that are not autocatalytic^14,37^.

Among these natural pathways, the Acetyl-CoA-Succinyl-CoA pathway family^3^ is a particularly interesting group. These include the reductive tricarboxylic acid cycle (rTCA), the dicarboxylate-4-hydroxybutyrate cycle (DC/4-HB), the 3-hydroxypropionate-4-hydroxybutyrate cycle (3-HP/4-HB), and the 3-hydroxypropionate bicycle (3-HP bicycle). This group of pathways shares a common structural feature: one half of the cycle converts Succinyl-CoA to Acetyl-CoA, while the other half catalyzes the reverse reaction. Furthermore, each of these pathways overlaps significantly with at least one other, making them highly relevant as templates for designing artificial pathways.

Their autocatalytic nature is another commonality, which is an important property of several biochemical pathways^14^. A reaction is autocatalytic if at least one reaction product is a catalyst in the reaction producing this product^38^. In an autocatalytic pathway, at least one metabolite, the autocatalyst, acts as a catalyst for its own formation, and together with external inputs, each cycle yields a net gain of this metabolite^14,37^. This autocatalyst needs to be present for the pathway to start up initially. These Acetyl-CoA-Succinyl-CoA pathways (e.g., rTCA, DC/4-HB, 3-HP/4-HB, 3-HP bicycle) were therefore central to the model due to their inherent autocatalysis and overlapping structures. The synthetic CETCH cycle^16^, which builds on these natural pathways, was also included, as well as proposed pathways from a paper by Bar-Even and his colleagues^26^. The Calvin-Benson-Basham (CBB) cycle^6^, despite being autocatalytic, was not included in the explored space due to its unique molecular context, which relies on recombination and carbohydrate chemistry, and lack of interaction with other cycles.

## The ILP Model for Finding Novel Artificial Pathways

**Fig. 6:**
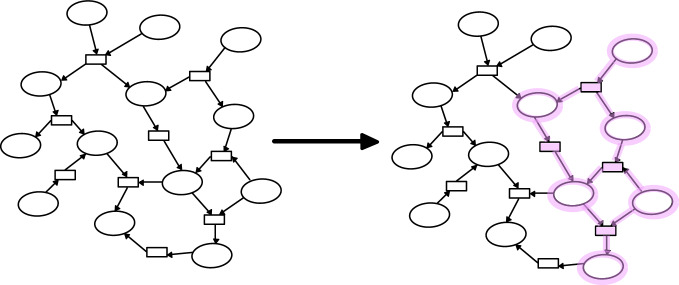
A flow query is a pathway search on a chemical reaction network. On the left is the network, with ovals representing molecules and rectangles representing reactions. The flow query is a set of constraints and an objective function which combined specifies desired structural aspects of the pathway searched for. A solution to a flow query is illustrated on the right.

To identify novel pathways within the modeled chemical space, we utilized integer linear programming (ILP), implemented within MØD as “flow queries”^20,37^. In this context, a pathway is seen as a hyperflow on the chemical reaction network, and a pathway query is therefore a flow query, with constraints on the flow, as visualized in Fig. 6.

These searches are built with objective functions and constraints as described below. The integer nature of the ILP model allows for integer values as flow solutions, which therefore represent only whole molecules in the solution space. Unlike classical flux balance analysis, which emphasizes continuous flux distributions under steady-state conditions^39^, the ILP approach with integer solution values focuses on the structure of a pathway, identifying which reactions are active rather than optimizing the flux magnitude (see ref.^20^ for a detailed comparison).

Defining the objective function focuses on the main driving force of this work, the optimization towards shorter artificial pathways. The main objective function is described in Equation (1). A description of the appearing variables and constants is given in Table 5. Shortest pathway searches can be achieved by minimizing the total use of edges in a solution, represented by *z*_*e*_. Additionally, to avoid scaled solutions with same edge numbers but up-scaled flow variable, *x*_*e*_ is minimized as well.

1 $$\min \left(\sum _{e\in E}{z}_{e}\cdot w+{x}_{e}\right)$$

2 $$0 < {x}_{v}^{in} < {x}_{v}^{out} < 2$$

**Table 5:** Variables and constants used in the ILP formulation

Symbol	Type	Description
*z*_*e*_	boolean variable	Indicator variable for flow on hyperedge *e*
*x*_*e*_	integer variable	Flow variable for a hyperedge *e*
*w*	constant	Weight to prioritize minimized edge usage, set to 1000

To find a hyperflow in the network, a set of sources and sinks needs to be defined. The sources are the molecules that we allow to have inflow, i.e., the molecules that can be put into the pathway. The sinks are the molecules that we allow to have outflow, i.e., the molecules that can be eventually produced by a pathway. In order to find an autocatalytic flow solution, only the autocatalytic molecule and the cofactors can be allowed as sources and sinks. In this way, the net reaction of the flow has only those molecules consumed and produced. The constraint in Equation (2) assures that the autocatalytic molecule *a* of the query has an input greater than zero and an output greater than the input, thereby defining the net production of at least one autocatalyst molecule. This modeling of autocatalysis follows the definition of autocatalysis presented in refs. ^20,37^.

The queries were formulated with objective functions tailored to specific optimization tasks, such as identifying the shortest autocatalytic cycles or finding alternative products. Flow queries were solved using the Gurobi optimizer^40^ (version 11.0.3) under an academic license. All computations were performed on a standard consumer laptop (AMD Ryzen 7 5700U, 16 GB RAM, Windows 11). The runtime for the search for 1000 solutions added up to just under 18 h.

The flow queries were performed on three molecules to represent the customizability of the search. Acetyl-CoA was chosen as a natural choice for a benchmark molecule because all of the included natural pathways have the overlap of this molecule in their pathways, while it also plays a role in many theoretical pathways^3,15,26^. Malate is a intermediate molecule in all of the included natural pathways as well as used as a product molecule for the CETCH cycle^16^. Propionyl-CoA was used as molecule that is only found in two of the natural pathways and a common benchmarking molecule in synthetic pathway design^16,17^.

## Evaluation of Pathway Solutions

Initially, the presence of the natural pathways in the solution space was evaluated using set operations. The intersection and difference between the natural pathway and the reaction space were calculated following the implementation of this approach^41^. The natural pathways were also confirmed by ILP search. Constraining the solution space to contain a specific core molecule from a natural pathway yielded the respective pathway as the solution.

Further, pathway solutions were manually inspected to identify interesting combinations. This step involved comparing key attributes of the proposed pathway. One of these key attributes is cofactor usage, which already gives a measure for energy consumption of a pathway and alignment with natural or proposed artificial pathways. Another one is the number of reaction steps, comparing the pathways length and efficiency in absolute length. Lastly, the involved reactions themselves were compared, determining how closely related a solution is to a known pathway in topology and what recombinations were proposed in the solution. This includes alternative reaction routes, as well as molecules not found in the natural space being used in the solutions. These evaluations provided insights into how each pathway diverges from natural pathways and how it mimics or extends known artificial pathways.

Thermodynamic feasibility was subsequently assessed to complement this qualitative analysis. Energies of formation for all molecules in the chemical space were calculated using the component-contribution method through the eQuilibrator 3.0 computational framework via the eQuilibrator API (version 0.6.0)^23,24,42,43^. The energies of formation were calculated at cellular conditions ${\Delta }_{f}{G}^{{\prime} \circ }$, with pH = 7 and ionic strength of 0.1 M. As a measure of feasibility the Gibbs free energy change ${\Delta }_{r}{G}^{{\prime} \circ }$ was calculated by post-annotating each flow solution with energies of formation for the overall reaction of the pathway. The sum of ${\Delta }_{f}{G}^{{\prime} \circ }$ of the reactants was subtracted from the sum of ${\Delta }_{f}{G}^{{\prime} \circ }$ of the products, as described in Equation (3). The calculation of the reaction energy provided a metric for the analysis of pathway favorability, and includes the thermodynamic activation cost required to create reactive molecules through ATP use.

3 $${\Delta }_{r}{G}^{{\prime} \circ }=\sum {\Delta }_{f}{G}_{products}^{{\prime} \circ }-\sum {\Delta }_{f}{G}_{reactants}^{{\prime} \circ }$$

Further insight into the biochemical feasibility was achieved by analyzing the cofactor usage. ATP and ADP were used as a measure for energy usage. The consumption of reduced redox cofactors, including NAD(P)H, Ubiquitin, and Ferredoxin, is a measure of the electrons needed for the pathway. Those two cofactor counts combined give an estimate of how expensive a pathway would be for an organism to perform. Those cofactors were counted in a given solution as part of the post-annotation analysis.

This combined approach of qualitative inspection and thermodynamic evaluation offered a framework for identifying and prioritizing both natural-like and novel artificial pathways for further investigation.

## Supplementary information

Supplementary Information

## Data Availability

The datasets generated and analyzed during the current study, as well as the underlying code are available in the GitHub repository https://github.com/anne-susann/C_fixation_pathway_design.
