# Supplementary material for: Computational approaches in chemical space exploration for carbon fixation pathways
Source: NPJ Syst Biol Appl. 2026 Jan 8;12:17. doi: 10.1038/s41540-025-00641-8 (PMC12868658; doi:10.1038/s41540-025-00641-8)
Supplement: Supplementary file 1 — Supplementary Information [file 41540_2025_641_MOESM1_ESM.pdf]

Supplementary Material - Computational Approaches in Chemical Space Exploration for Carbon Fixation Pathways

Anne-Susann Abel, Nino Lauber, Jakob Lykke Andersen, Rolf Fagerberg, Daniel Merkle, and Christoph Flamm

Table S1: Collection of the 49 initial molecules for the chemical space expansion, with their molecule name as well as their input format. Some molecules have abstract labels like [CoA] instead of atom labels for ease of use. COA: coenzyme A, 3HP: 3-hydroxypropionic acid , 4HB: 4-hydroxybutyric acid, PEP: phosphoenolpyruvic acid, GAP: glyceraldehyde-3-phosphate, DHAP: dihydroxyacetone phosphate.

**Molecule Name**	**Smiles Representation**
SUCCINYL COA	OC(=O)CCC(=O)S[CoA]
SUCCINATE SEMIALDEHYDE	O=CCCC(=O)O
4HB	C(CC(=O)O)CO
4-HYDROXYBUTYRYL COA	C(CC(=O)S[CoA])CO
CROTONYL-COA	CC=CC(=O)S[CoA]
3-HYDROXYBUTYRYL COA	CC(CC(=O)S[CoA])O
ACETOACETYL COA	CC(=O)CC(=O)S[CoA]
ACETYL COA	CC(=O)S[CoA]
PYRUVATE	CC(=O)C(=O)O
PEP	C=C(C(=O)O)OP(=O)(O)O
OXALACETIC ACID	C(C(=O)C(=O)O)C(=O)O
MALIC ACID	C(C(C(=O)O)O)C(=O)O
FUMARIC ACID	C(=CC(=O)O)C(=O)O

SUCCINIC ACID	OC(=O)CCC(=O)O
MALONYL COA	C(C(=O)O)C(=O)S[CoA]
MALONATE SEMIALDEHYDE	OC(=O)CC=O
3HP	C(CO)C(=O)O
3-HYDROXYPROPIONATE COA	C(CO)C(=O)S[CoA]
ACRYLYL COA	C=CC(=O)S[CoA]
METHYLMALONYL COA	CC(C(=O)O)C(=O)S[CoA]
2-KETOGLUTARIC ACID	C(CC(=O)O)C(=O)C(=O)O
ISOCITRIC ACID	C(C(C(C(=O)O)O)C(=O)O)C(=O)O
ACONITIC ACID	C(C(=CC(=O)O)C(=O)O)C(=O)O
CITRIC ACID	C(C(=O)O)C(CC(=O)O)(C(=O)O)O
CITRYL COA	C(C(=O)O)C(CC(=O)S[CoA])(C(=O)O)O
MALYL COA	C(C(C(=O)O)O)C(=O)S[CoA]
GLYOXYLATE	C(=O)C(=O)O
PROPIONYL COA	CCC(=O)S[CoA]
METHYLMALYL COA	CC(C(C(=O)O)O)C(=O)S[CoA]
MESACONYL C1 COA	CC(=CC(=O)O)C(=O)S[CoA]
MESACONYL C4 COA	CC(=CC(=O)S[CoA])C(=O)O
CITRAMALYL COA	CC(CC(=O)S[CoA])(C(=O)O)O
ETHYLMALONYL COA	CCC(C(=O)O)C(=O)S[CoA]
METHYLSUCCINYL COA	OC(=O)CC(C)C(=O)S[CoA]
RIBULOSE-1_5-BISPHOSPHATE	C(C(C(C(=O)COP(=O)(O)O)O)O)OP(=O)(O)O
3-PHOSPHOGLYCERATE	C(C(C(=O)O)O)OP(=O)(O)O
1_3-BISPHOSPHOGLYCERATE	C(C(C(=O)OP(=O)(O)O)O)OP(=O)(O)O
GAP	C(C(C=O)O)OP(=O)(O)O
DHAP	C(C(=O)COP(=O)(O)O)O

FRUCTOSE-1_6-BISPHOSPHATE	C(C(C(C(C(C(=O)COP(=O)(O)O)O)O)O)OP(=O)(O)O
FRUCTOSE-6-P	C(C(C(C(C(C(=O)CO)O)O)O)OP(=O)(O)O
ERYTHROSE-4-P	C(C(C(C=O)O)O)OP(=O)(O)O
RIBULOSE-5-P	C(C(C(C(=O)CO)O)O)OP(=O)(O)O
XYLULOSE-5-P	C(C(C(C(=O)CO)[xyO][H])O)OP(=O)(O)O
SEDOHEPTULOSE-1_7-BISPHOSPHATE	C(C(C(C(C(C(C(=O)COP(=O)(O)O)O)O)O)O)O)OP(=O)(O)O
SEDOHEPTULOSE-7-PHOSPHATE	C(C(C(C(C(C(C(=O)CO)O)O)O)O)O)OP(=O)(O)O
RIBOSE-5-PHOSPHATE	C(C(C(C(C=O)O)O)O)OP(=O)(O)O
beta-ALANINE	C(CN)C(=O)O
L-ALANINE	CC(C(=O)O)N

Table S2: Collection of the 20 cofactors used for the chemical space expansion, with their molecule name as well as their input format. Most molecules have abstract labels like [NADH] instead of atom labels for ease of use. CoASH: coenzyme A unbound form, Pi: phosphate, PPi: pyrophosphate, UQred: ubiquitin reduced, UQox: ubiquitin oxidised, Fdred: ferredoxin reduced, Fdox: ferredoxin oxidised.

**Molecule Name**	**Smiles Representation**
CoASH	[CoA][S][H]
ATP	[Ad]OP(=O)(O)OP(=O)(O)OP(=O)(O)O
ADP	[Ad]OP(=O)(O)OP(=O)(O)O
AMP	[Ad]OP(=O)(O)O
Pi	OP(=O)(O)O
PPi	OP(=O)(O)OP(=O)(O)O
NADPH	[NADP][H]
NADP+	[NADP+]
NADH	[NAD][H]
NAD+	[NAD+]
Hplus	[H+]
H2O	O
HCO3-	C(=O)(O)[O-]
CO2	C(=O)=O
UQred	O=[UQ]=O
UQox	O[UQ]O
Fdred	[Fdred]
Fdox	[Fdox]
O2	O=O
H2O2	OO

Figure S1: Collection of all graph transformation rules used in the creation of the chemical space on the following 16 pages, including an overview list. The rules are depicted as double pushout diagrams. (L) shows the left side of the reaction, (R) shows the right side of the reaction, and (K) represents the reaction core, with the bonds and labels that are not changing during the reaction. Where applicable, labels like (_X), (_Y), and (_Z), are listed underneath the diagrams with the atom types they can represent. Some labels represent whole cofactors instead of single atoms: NADP(+), NAD(+), UQ: Ubiquitin, Fdox: Ferredoxin oxidised, Fdred: Ferredoxin reduced, CoA: Coenzyme A, Ad: Adenine. The names of the rules are the names of the original enzymes that the rules were modeled after and modified.

Contents

0.1 DPO Diagramms of Rule(s) 3
0.1.1 rTCA_activation_succinyl_CoA_synthetase_01 3
0.1.2 rTCA_activation_ATP_citrate_lyase_01 3
0.1.3 rTCA_reduction_malate_dehydrogenase_01 3
0.1.4 rTCA_reduction_fumarate_reductase_01_2 4
0.1.5 rTCA_dehydration_fumarase_01 4
0.1.6 rTCA_dehydration_rehydration_aconitase_02 4
0.1.7 rTCA_dehydration_rehydration_crotonase_02_1 5
0.1.8 rTCA_carboxylation_2KFOR_PFOR_01 5
0.1.9 rTCA_carboxylation_IDH_01 5
0.1.10 rTCA_carboxylation_PEPC_01_1 6
0.1.11 rTCA_activation_dikinase_01_1 6
0.1.12 3HP4HB_dehydration_4HBCoA_dehydratase_01 6
0.1.13 3HP4HB_carboxylation_acetyl-CoA_carboxylase_01 7
0.1.14 3HP4HB_rearrangement_methylmalonyl-CoA_mutase_01 7
0.1.15 3HP4HB_reduction_acrylyl-CoA_reductase_01 7
0.1.16 3HP4HB_oxidation_3-hydroxybutyryl-CoA_dehydrogenase_01 8
0.1.17 3HP4HB_dehydration_3HPCoA_dehydratase_01 8
0.1.18 3HP4HB_reduction_succinyl-CoA_reductase_01 8
0.1.19 3HP4HB_reduction_3-hydroxypropionate_dehydrogenase_01 9
0.1.20 3HP4HB_activation_4HBCoA_synthetase_01 9
0.1.21 3HP4HB_transferase_acetyl-CoA_C-acyltransferase_01_1 9
0.1.22 3HPbicy_mesaconyl_C1_C4_CoA_transferase_01 10
0.1.23 3HPbicy_activation_methylmalyl-CoA_lyase_01 10
0.1.24 3HPbicy_transferase_succinyl-CoA_malate-CoA_transferase_01 10
0.1.25 3HPbicy_oxidation_succinate_dehydrogenase_01 11
0.1.26 3HPbicy_oxidation_succinate_dehydrogenase_01_1 11
0.1.27 3HPbicy_oxidation_succinate_dehydrogenase_01_2 11
0.1.28 3HPbicy_transferase_succinyl-CoA_malate-CoA_transferase_a_01 12
0.1.29 3HPbicy_transferase_succinyl-CoA_malate-CoA_transferase_b_01 12
0.1.30 CETCH_activation_methylmalyl-CoA_lyase_01 12
0.1.31 CETCH_activation_lysis_malate_synthase_02 13
0.1.32 CETCH_carboxylation_ccr_carboxylase/reductase_01 13
0.1.33 CETCH_dehydration_rehydration_mesCoA_hydratase_02_2 13
0.1.34 CETCH_oxidation_propionyl-CoA/methylsuccinyl-CoA_oxidase_01 14
0.1.35 CETCH_rearrangement_ethylmalonyl-CoA_mutase_02 14
0.1.36 GLYOX_lysis_isocitrate_lyase_01 14
0.1.37 GLYOX_activation_malate_synthase_a_01 15
0.1.38 GLYOX_lysis_isocitrate_lyase_02_reverse 15
0.1.39 GLYOX_alanine_2,3_aminomutase_01 15
0.1.40 GLYOX_transaminase_01 16

0.1.41 GLYOX__transaminase__01 16
0.1.42 GLYOX__methylmalonyl-CoA__carboxytransferase__01 16

0.1 DPO Diagramms of Rule(s)

0.1.1 rTCA__activation__succinyl__CoA__synthetase__01

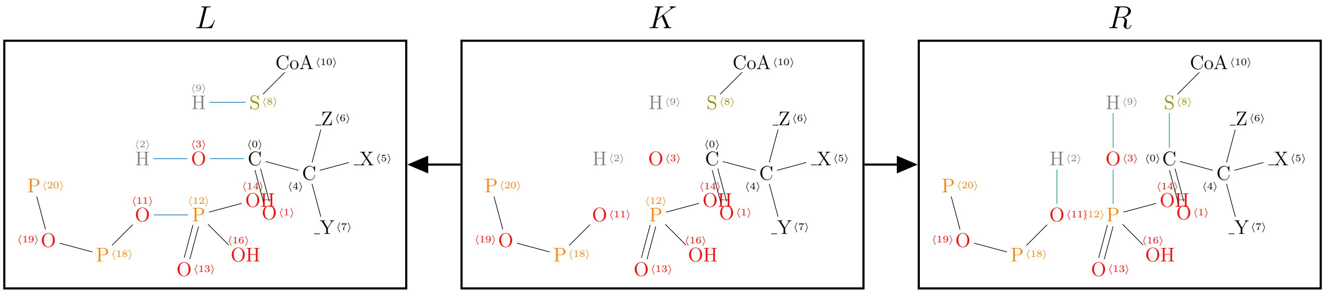

Files: out/001__r__0__11300110__{L, K, R}

_X ∈ {‘C’, ‘H’}
_Y ∈ {‘C’, ‘H’}
_Z ∈ {‘C’, ‘H’}

0.1.2 rTCA__activation__ATP__citrate__lyase__01

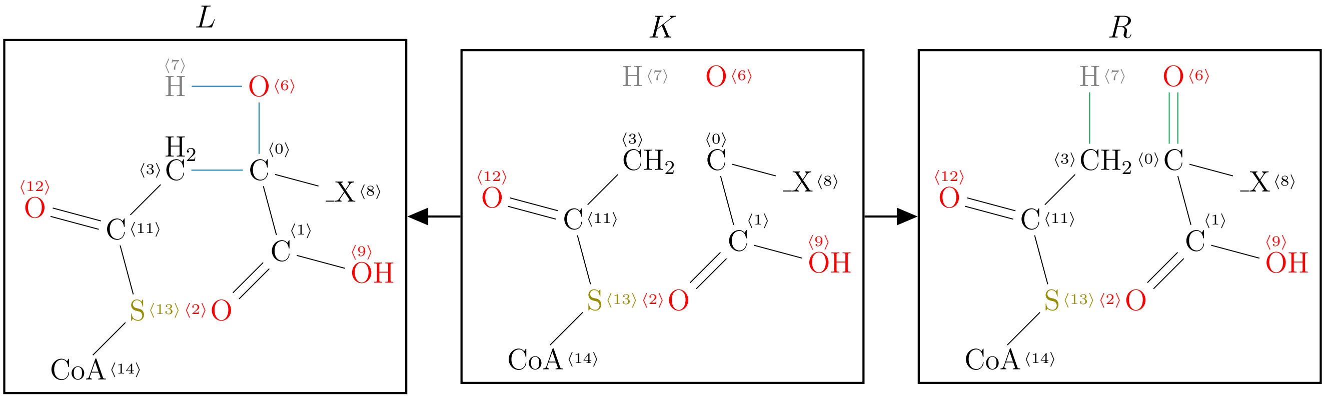

Files: out/004__r__1__11300110__{L, K, R}

_X ∈ {‘C’, ‘H’}

0.1.3 rTCA__reduction__malate__dehydrogenase__01

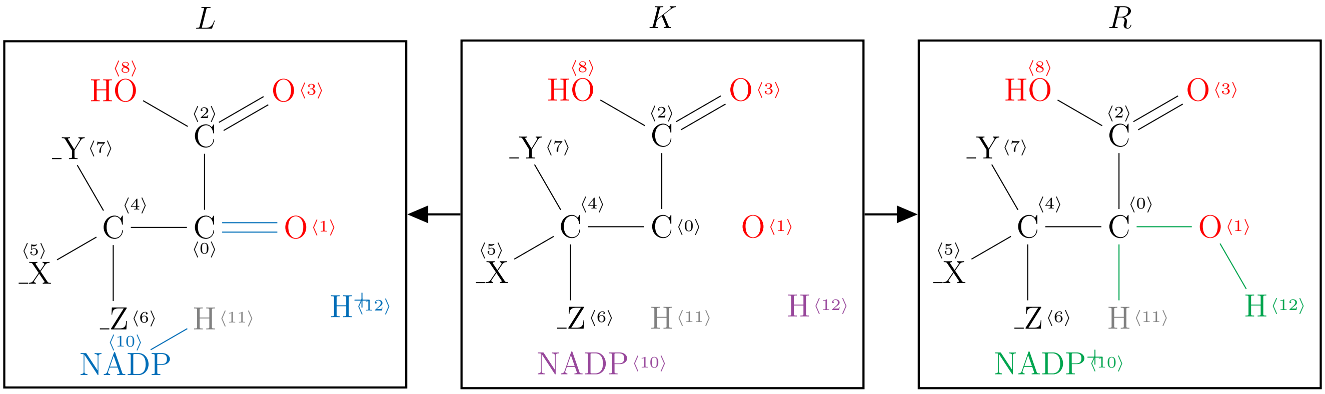

Files: out/007__r__2__11300110__{L, K, R}

_X ∈ {‘C’, ‘H’}
_Y ∈ {‘C’, ‘H’}
_Z ∈ {‘C’, ‘H’}

0.1.4 rTCA_reduction_fumarate_reductase_01_2

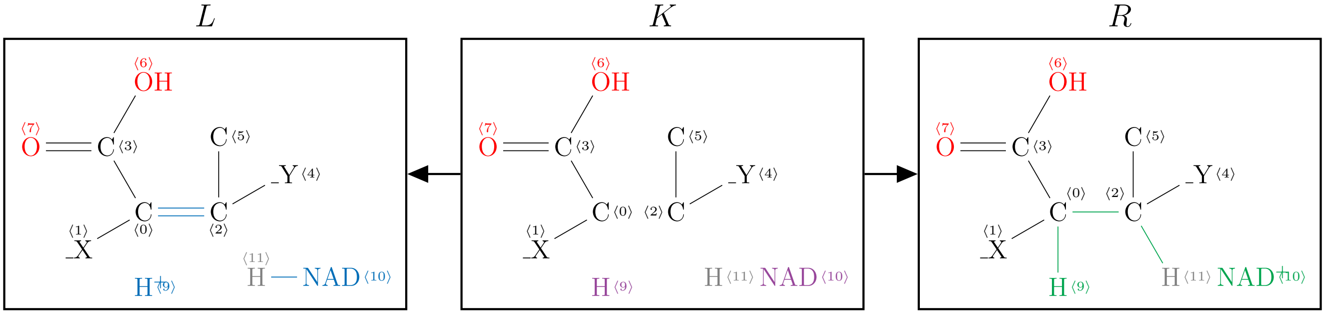

Files: out/010_r_5_11300110_{L, K, R}

_X ∈ {'H', 'C'}
_Y ∈ {'H', 'C'}

0.1.5 rTCA_dehydration_fumarase_01

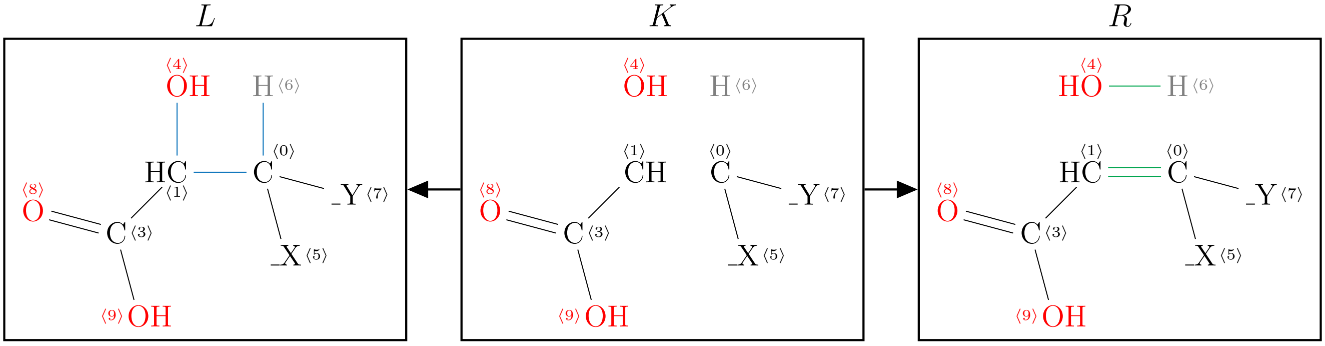

Files: out/013_r_6_11300110_{L, K, R}

_X ∈ {'C', 'H'}
_Y ∈ {'C', 'H'}

0.1.6 rTCA_dehydration_rehydration_aconitase_02

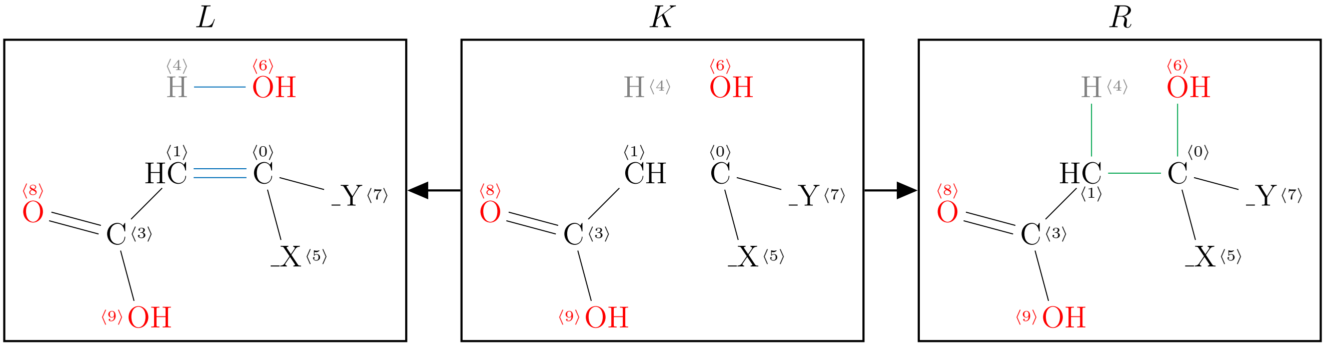

Files: out/016_r_7_11300110_{L, K, R}

_X ∈ {'C'}
_Y ∈ {'C'}

0.1.7 rTCA_dehydration_rehydration_crotonase_02_1

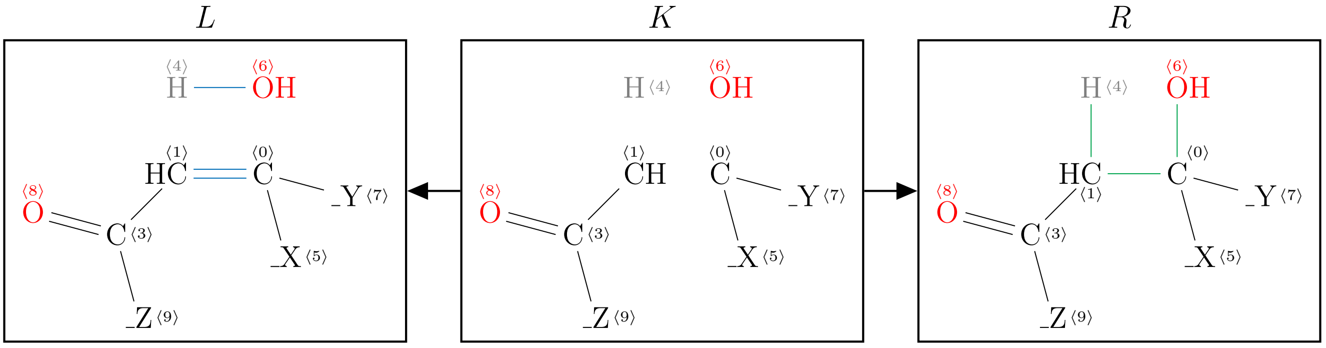

Files: out/019_r_8_11300110_{L, K, R}

_X ∈ {'C', 'H'}
_Y ∈ {'C', 'H'}
_Z ∈ {'O', 'S'}

0.1.8 rTCA_carboxylation_2KFOR_PFOR_01

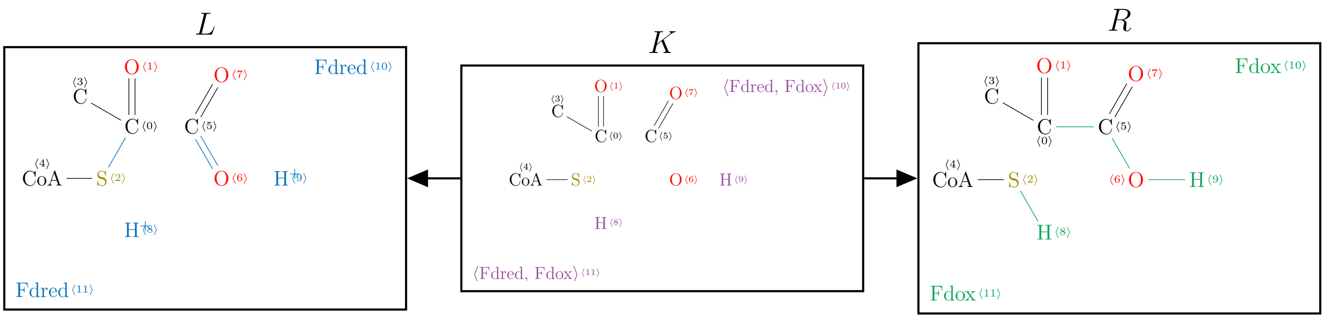

Files: out/022_r_9_11300110_{L, K, R}

0.1.9 rTCA_carboxylation_IDH_01

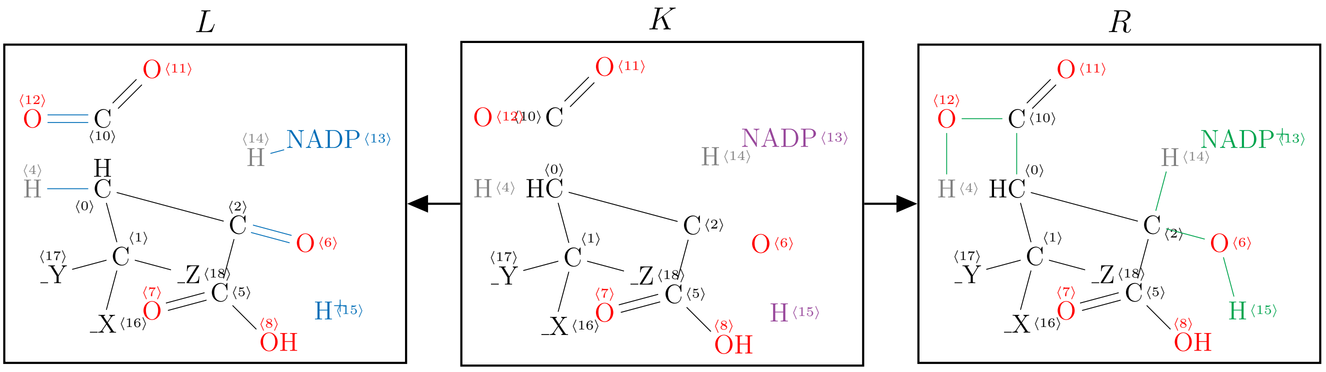

Files: out/025_r_10_11300110_{L, K, R}

_X ∈ {'C', 'H'}
_Y ∈ {'C', 'H'}
_Z ∈ {'C', 'H'}

0.1.10 rTCA_carboxylation_PEPC_01_1

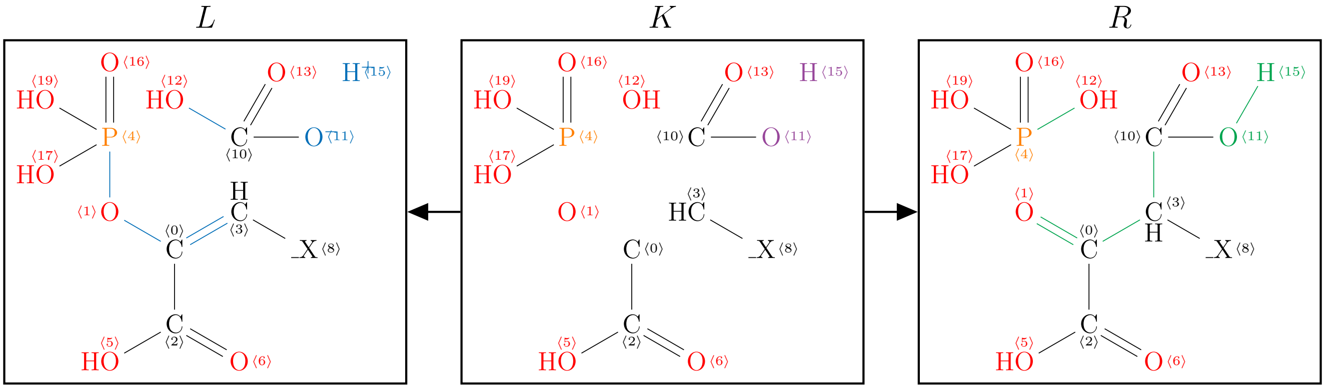

Files: out/028_r_12_11300110_{L, K, R}

_X ∈ {'H', 'C'}

0.1.11 rTCA_activation_dikinase_01_1

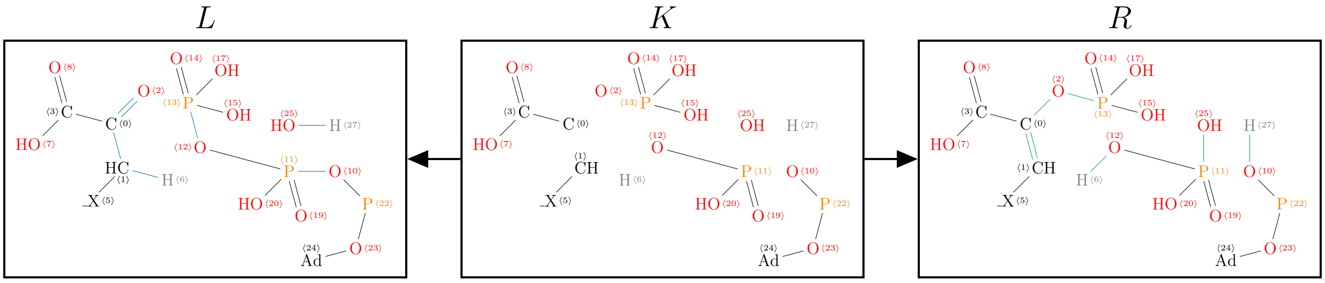

Files: out/031_r_14_11300110_{L, K, R}

_X ∈ {'H', 'C'}

0.1.12 3HP4HB_dehydration_4HBCoA_dehydratase_01

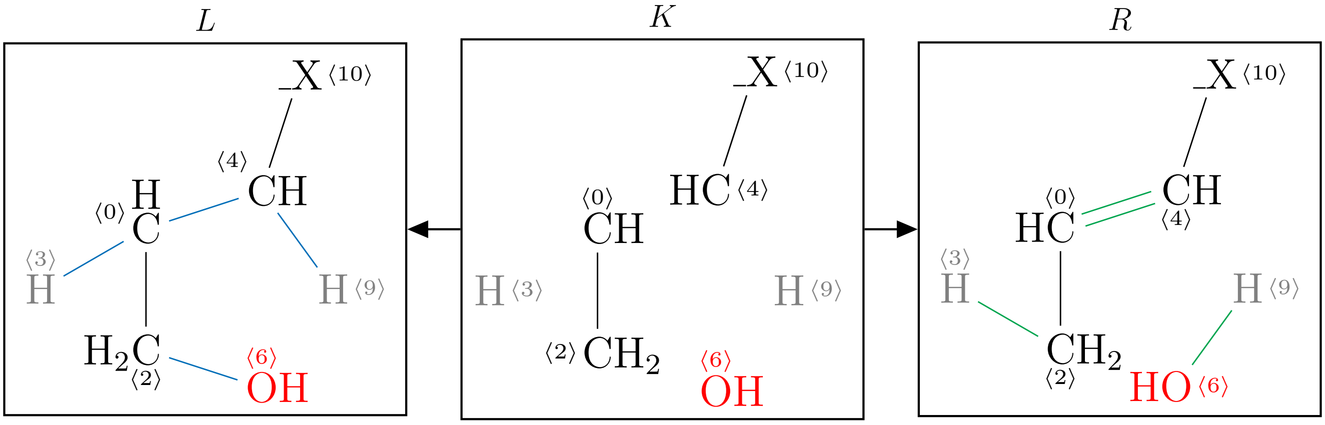

Files: out/034_r_15_11300110_{L, K, R}

_X ∈ {'C'}

0.1.13 3HP4HB__carboxylation__acetyl-CoA__carboxylase__01

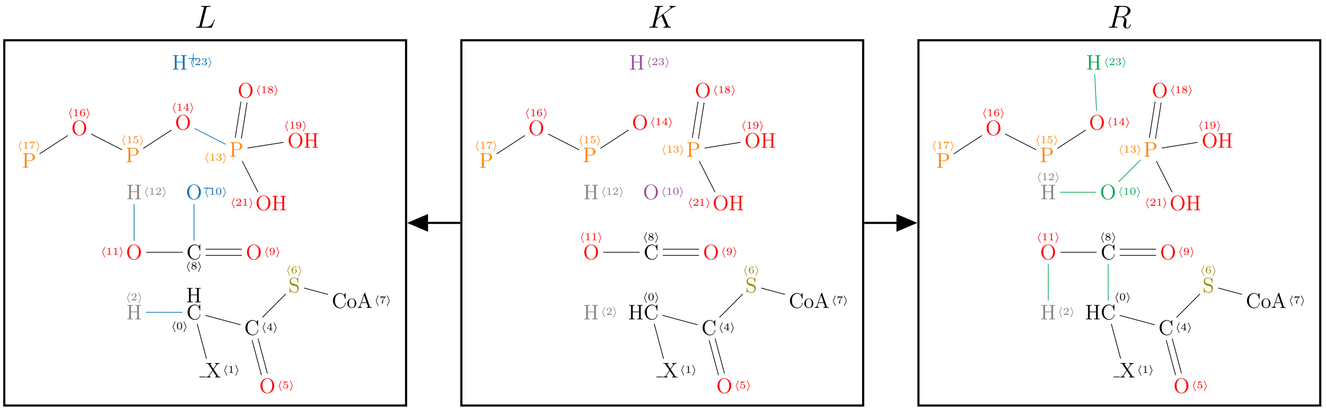

Files: out/037__r__16__11300110__{L, K, R}

_X ∈ {‘C’, ‘H’}

0.1.14 3HP4HB__rearrangement__methylmalonyl-CoA__mutase__01

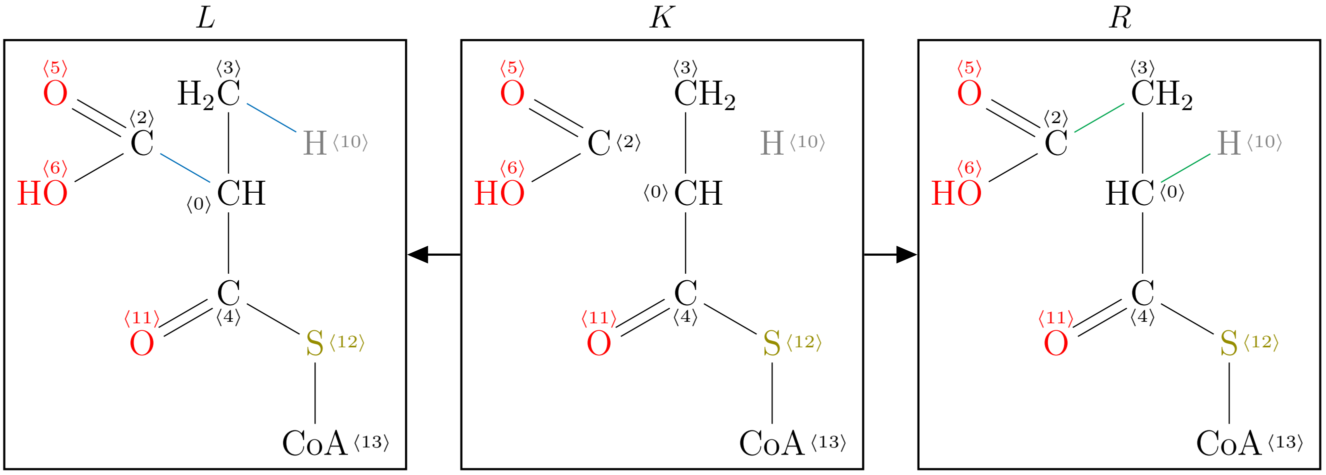

Files: out/040__r__17__11300110__{L, K, R}

0.1.15 3HP4HB__reduction__acrylyl-CoA__reductase__01

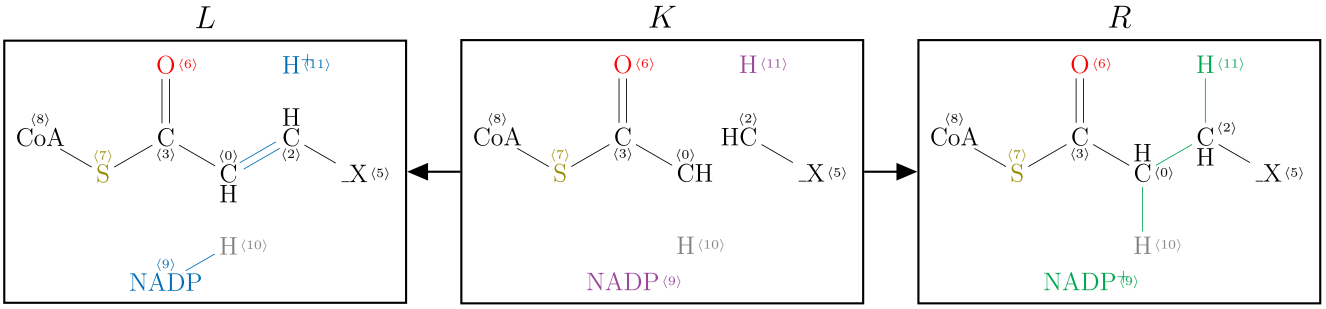

Files: out/043__r__18__11300110__{L, K, R}

_X ∈ {‘C’, ‘H’}

0.1.16 3HP4HB_oxidation_3-hydroxybutyryl-CoA_dehydrogenase_01

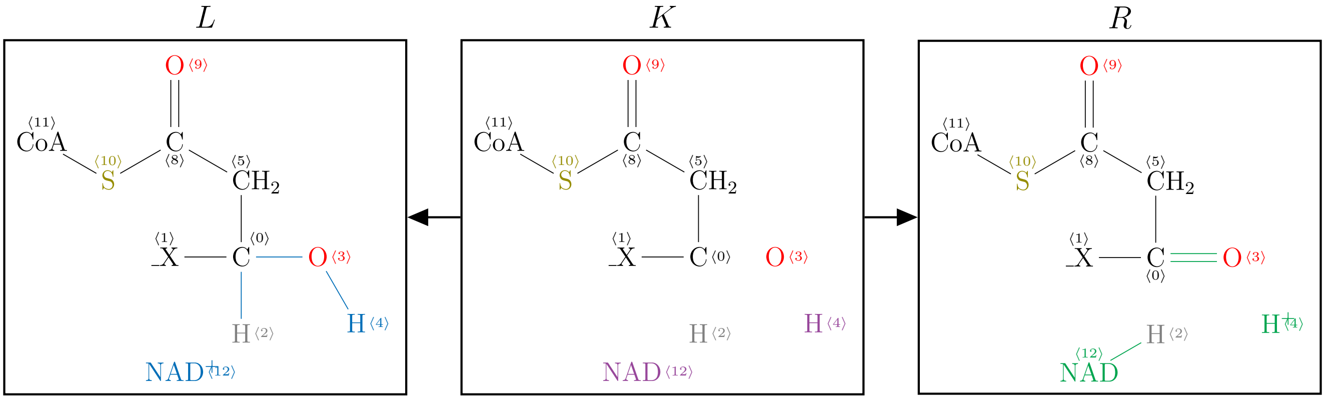

Files: out/046_r_19_11300110_{L, K, R}

_X ∈ {'C', 'H'}

0.1.17 3HP4HB_dehydration_3HPCoA_dehydratase_01

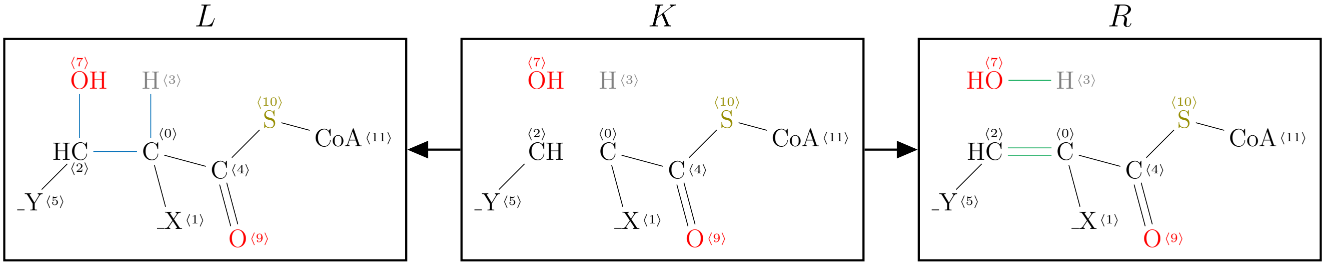

Files: out/049_r_20_11300110_{L, K, R}

_X ∈ {'C', 'H'}
_Y ∈ {'C', 'H'}

0.1.18 3HP4HB_reduction_succinyl-CoA_reductase_01

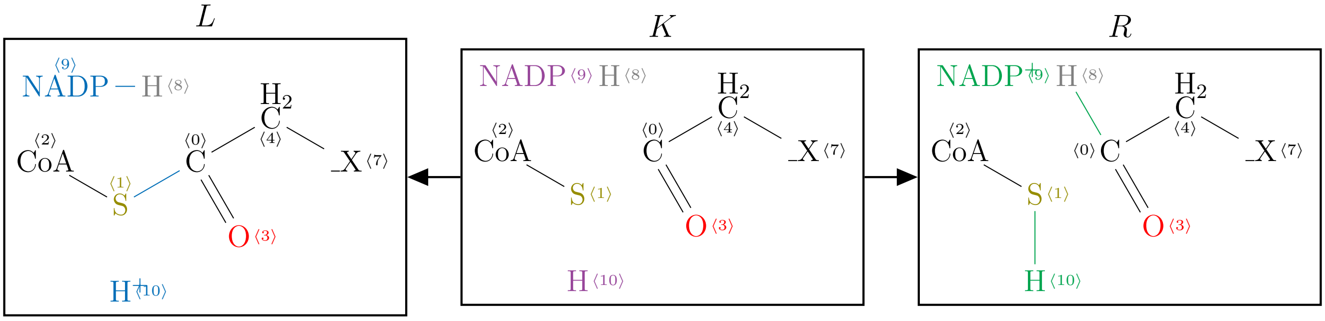

Files: out/052_r_21_11300110_{L, K, R}

_X ∈ {'C'}

0.1.19 3HP4HB_reduction_3-hydroxypropionate_dehydrogenase_01

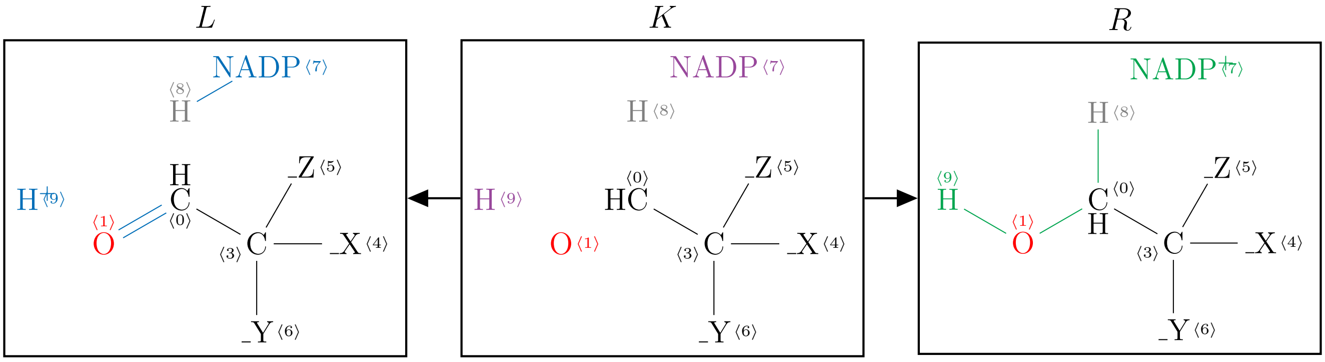

Files: out/055_r_22_11300110_{L, K, R}

_X ∈ {'C', 'H'}
_Y ∈ {'C', 'H'}
_Z ∈ {'C', 'H'}

0.1.20 3HP4HB_activation_4HBCoA_synthetase_01

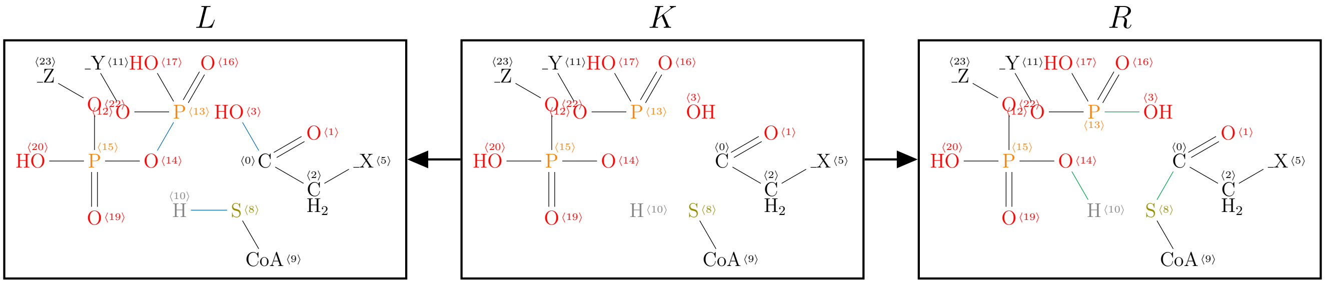

Files: out/058_r_23_11300110_{L, K, R}

_X ∈ {'C'}
_Y ∈ {'H', 'P'}
_Z ∈ {'P', 'Ad'}

0.1.21 3HP4HB_transferase_acetyl-CoA_C-acyltransferase_01_1

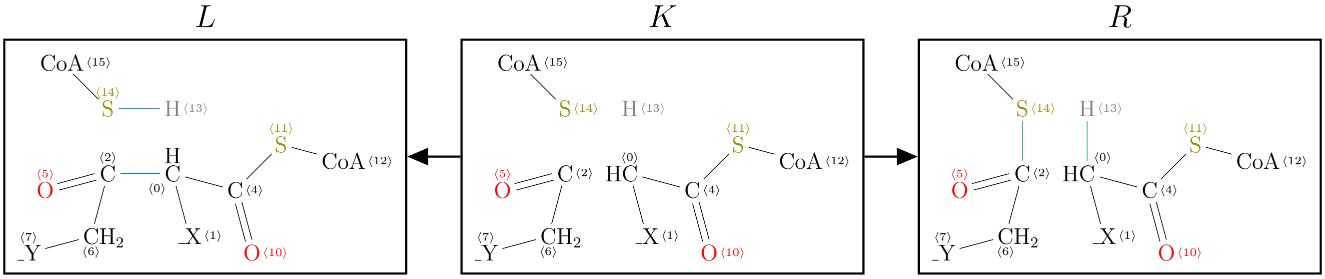

Files: out/061_r_25_11300110_{L, K, R}

_X ∈ {'H', 'C'}
_Y ∈ {'H', 'C'}

0.1.22 3HPbicy_mesaconyl_C1_C4_CoA_transferase_01

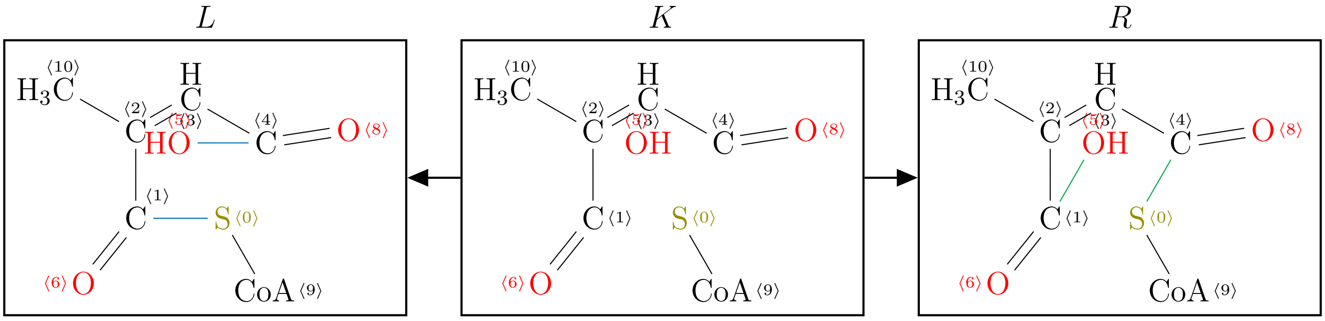

Files: out/064_r_26_11300110_{L, K, R}

0.1.23 3HPbicy_activation_methylmalyl-CoA_lyase_01

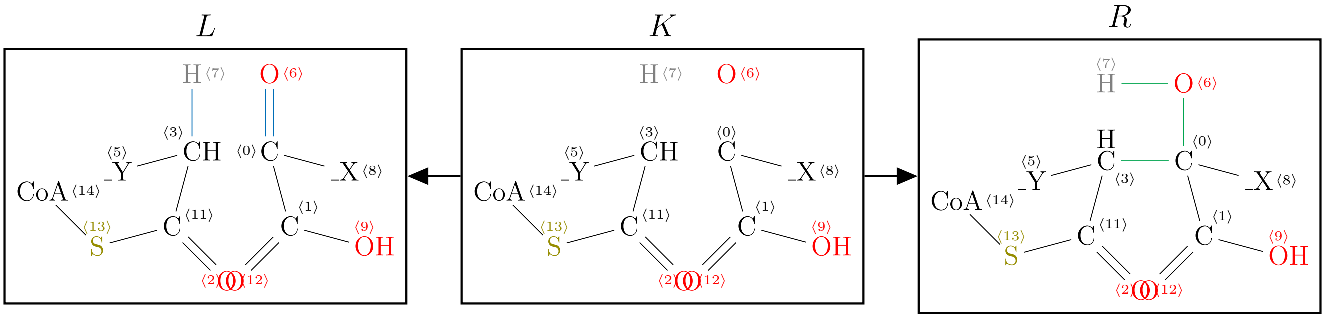

Files: out/067_r_27_11300110_{L, K, R}

`_X` ∈ {'C', 'H'}
`_Y` ∈ {'C', 'H'}

0.1.24 3HPbicy_transferase_succinyl-CoA_malate-CoA_transferase_01

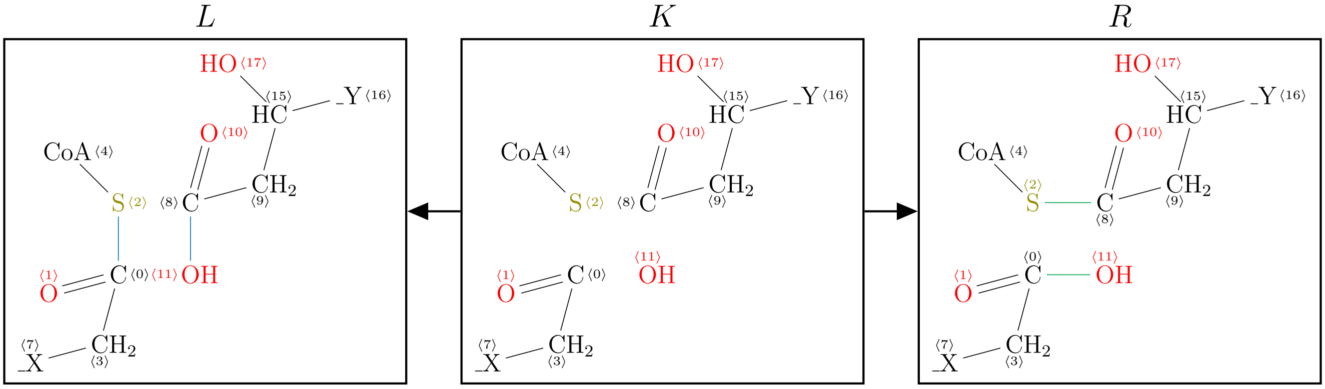

Files: out/070_r_28_11300110_{L, K, R}

`_X` ∈ {'C'}
`_Y` ∈ {'C'}

0.1.25 3HPbicy_oxidation_succinate_dehydrogenase_01

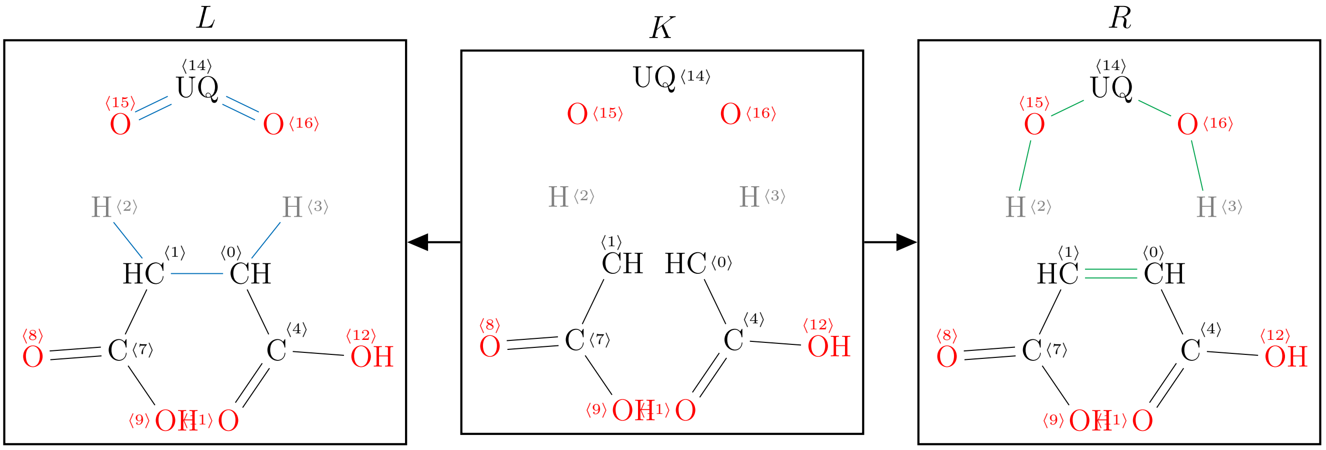

Files: out/073_r_29_11300110_{L, K, R}

0.1.26 3HPbicy_oxidation_succinate_dehydrogenase_01_1

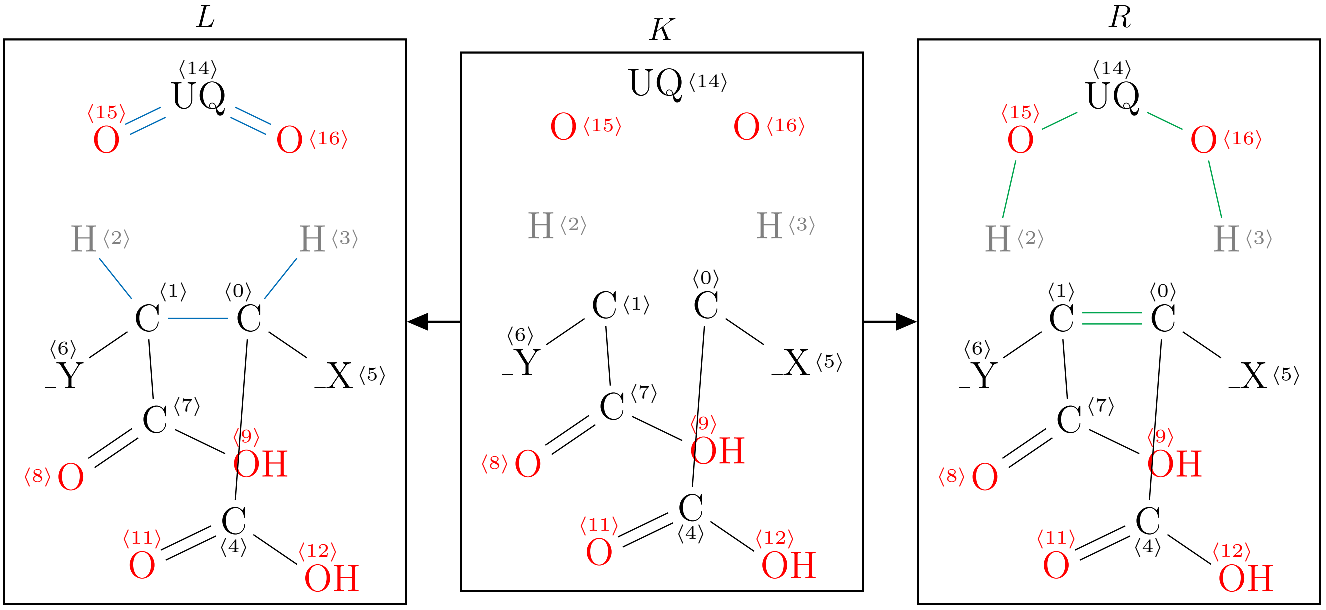

Files: out/076_r_30_11300110_{L, K, R}

`_X` ∈ {'`H`', '`C`'}
`_Y` ∈ {'`H`', '`C`'}

0.1.27 3HPbicy_oxidation_succinate_dehydrogenase_01_2

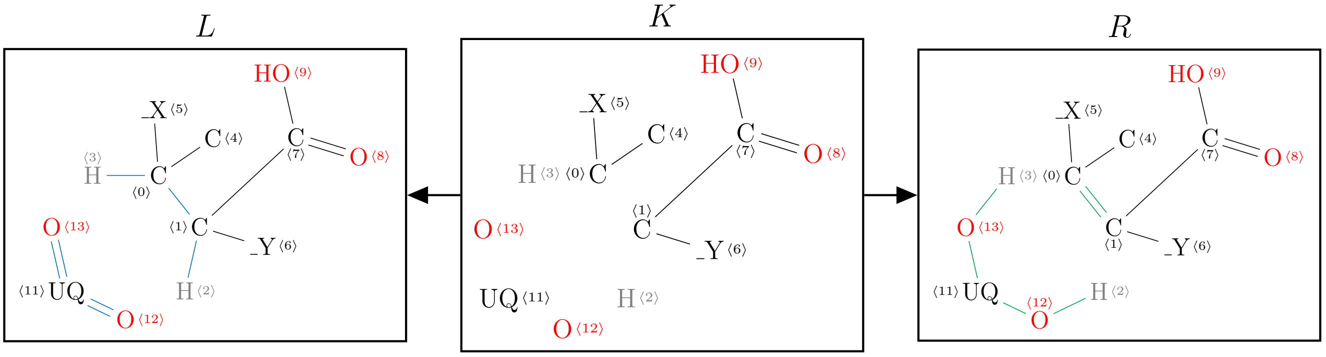

Files: out/079_r_31_11300110_{L, K, R}

_X ∈ {'H', 'C'}
_Y ∈ {'H', 'C'}

0.1.28 3HPbicy__transferase__succinyl-CoA__malate-CoA__transferase__a__01

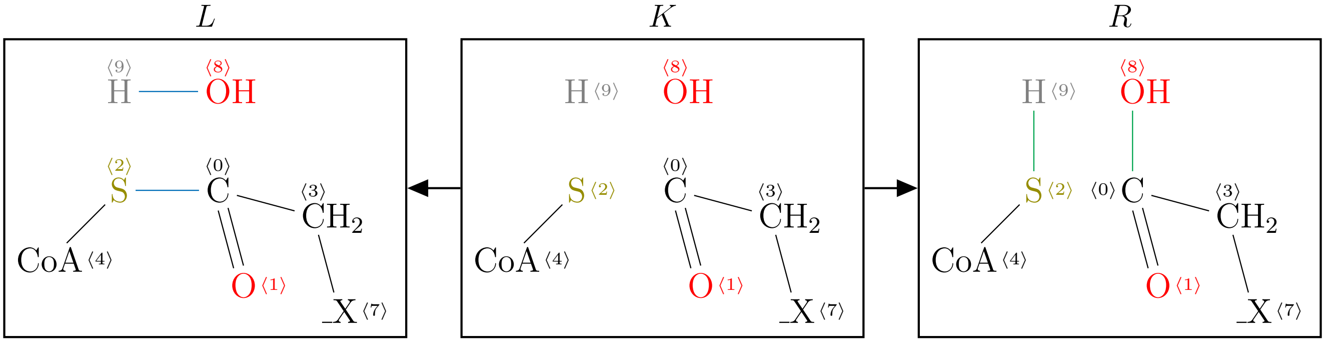

Files: out/082__r__33__11300110__{L, K, R}

_X ∈ {'H', 'C'}

0.1.29 3HPbicy__transferase__succinyl-CoA__malate-CoA__transferase__b__01

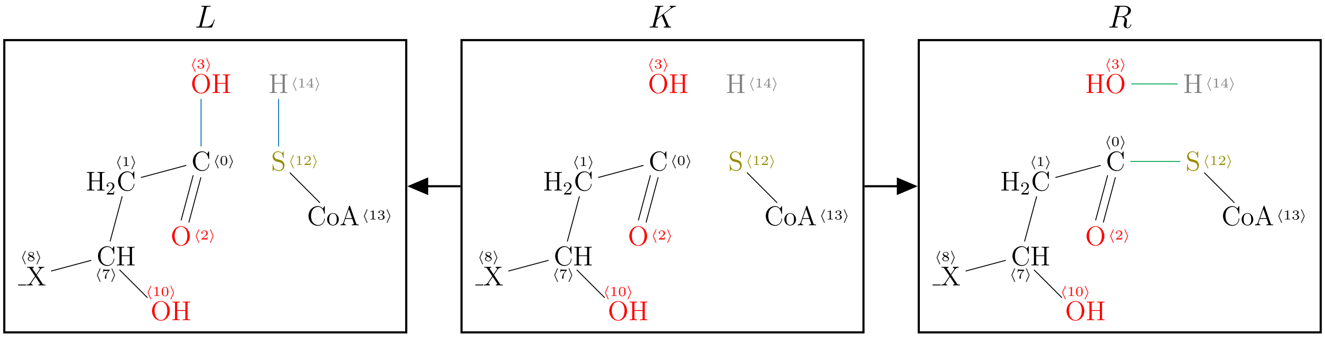

Files: out/085__r__35__11300110__{L, K, R}

_X ∈ {'H', 'C'}

0.1.30 CETCH__activation__methylmalyl-CoA__lyase__01

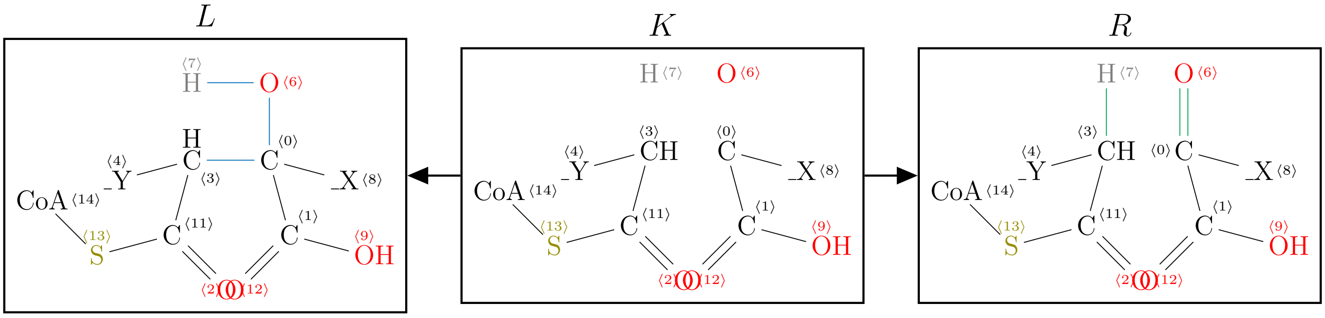

Files: out/088__r__46__11300110__{L, K, R}

_Y ∈ {'C', 'H'}
_X ∈ {'C', 'H'}

0.1.31 CETCH_activation_lysis_malate_synthase_02

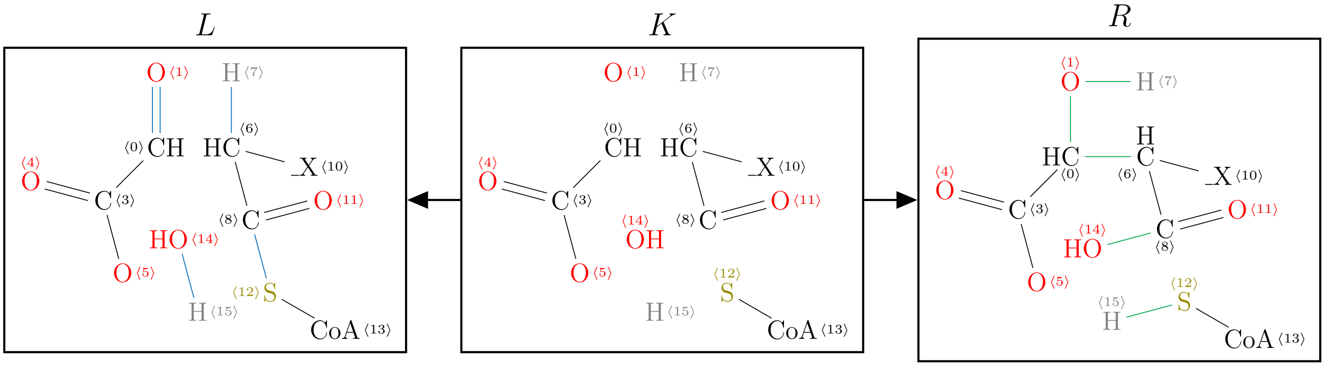

Files: out/091_r_47_11300110_{L, K, R}

_X ∈ {'C', 'H'}

0.1.32 CETCH_carboxylation_ccr_carboxylase/reductase_01

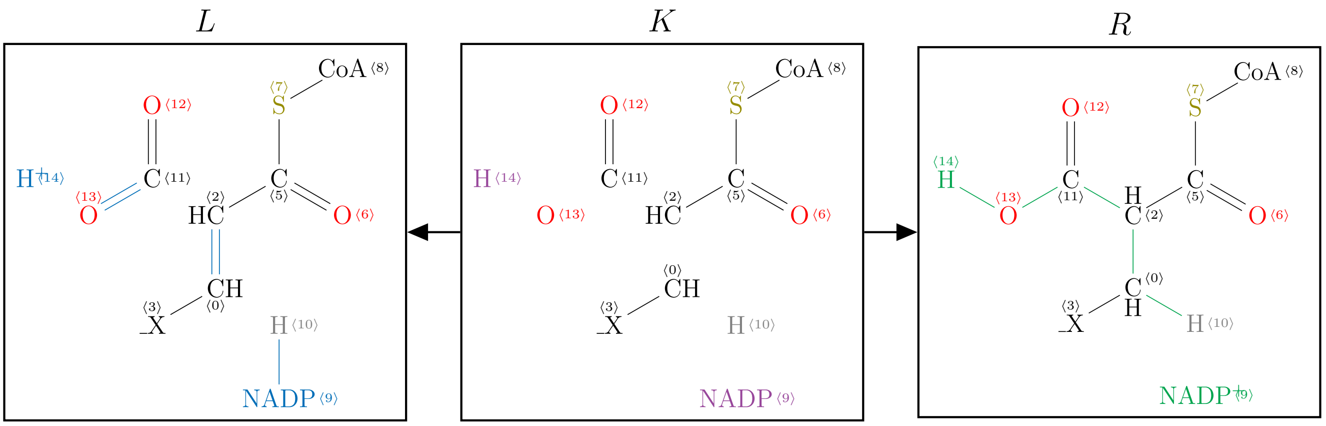

Files: out/094_r_48_11300110_{L, K, R}

_X ∈ {'C', 'H'}

0.1.33 CETCH_dehydration_rehydration_mesCoA_hydratase_02_2

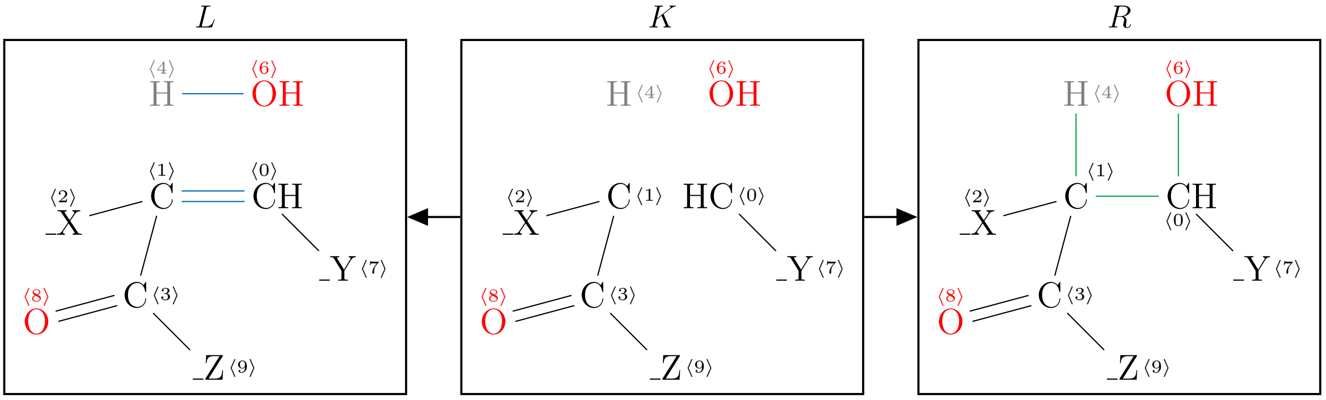

Files: out/097_r_49_11300110_{L, K, R}

_X ∈ {'C', 'H'}
_Y ∈ {'C', 'H'}
_Z ∈ {'O', 'S'}

0.1.34 CETCH_oxidation_propionyl-CoA/methylsuccinyl-CoA_oxidase_01

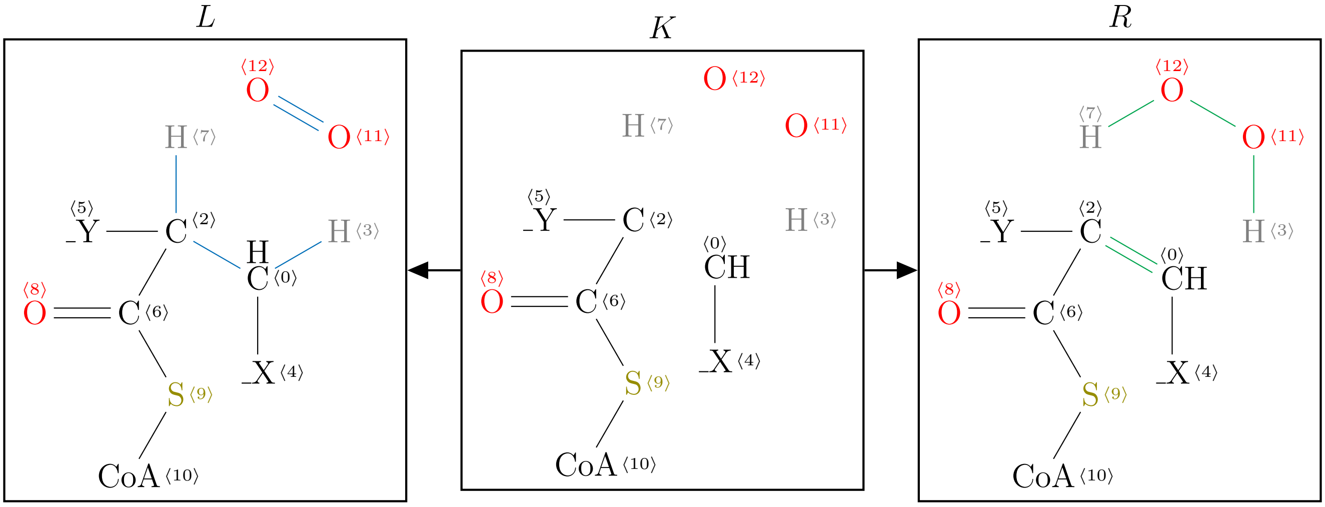

Files: out/100_r_50_11300110_{L, K, R}

_X ∈ {‘C’, ‘H’}
_Y ∈ {‘C’, ‘H’}

0.1.35 CETCH_rearrangement_ethylmalonyl-CoA_mutase_02

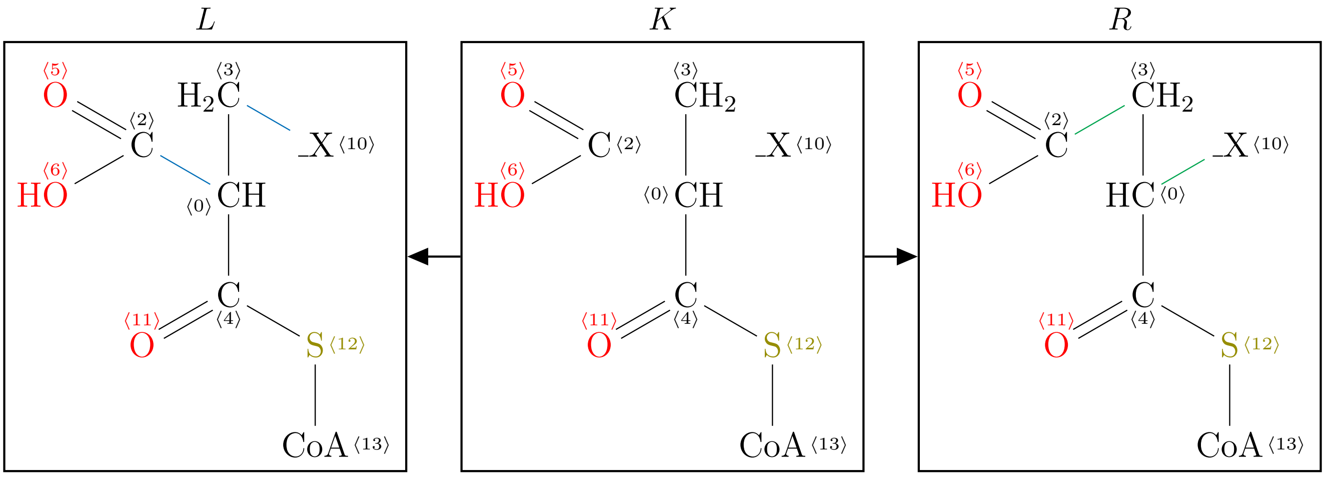

Files: out/103_r_51_11300110_{L, K, R}

_X ∈ {‘C’, ‘H’}

0.1.36 GLYOX_lysis_isocitrate_lyase_01

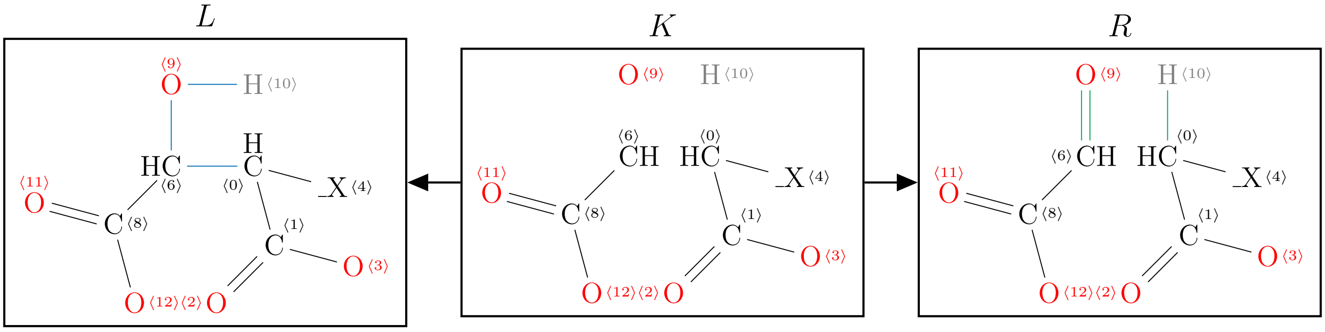

Files: out/106_r_52_11300110_{L, K, R}

_X ∈ {‘H’, ‘C’}

0.1.37 GLYOX_activation_malate_synthase_a_01

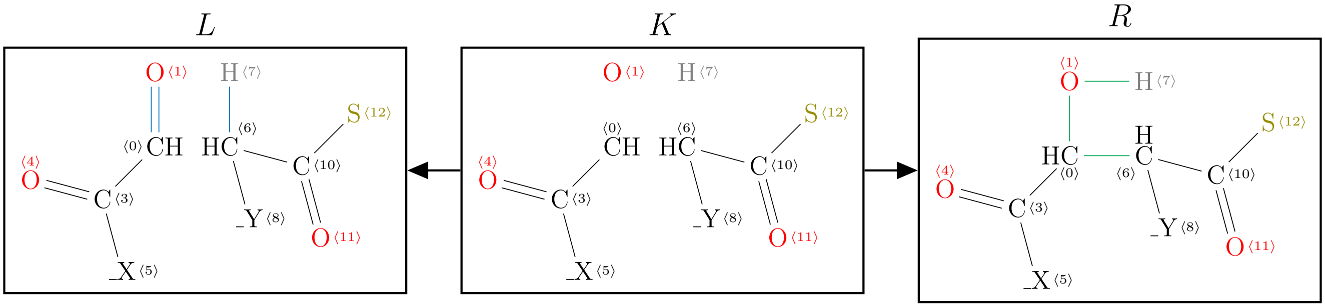

Files: out/109_r_53_11300110_{L, K, R}

_X ∈ {'O', 'S'}
_Y ∈ {'C', 'H'}

0.1.38 GLYOX_lysis_isocitrate_lyase_02_reverse

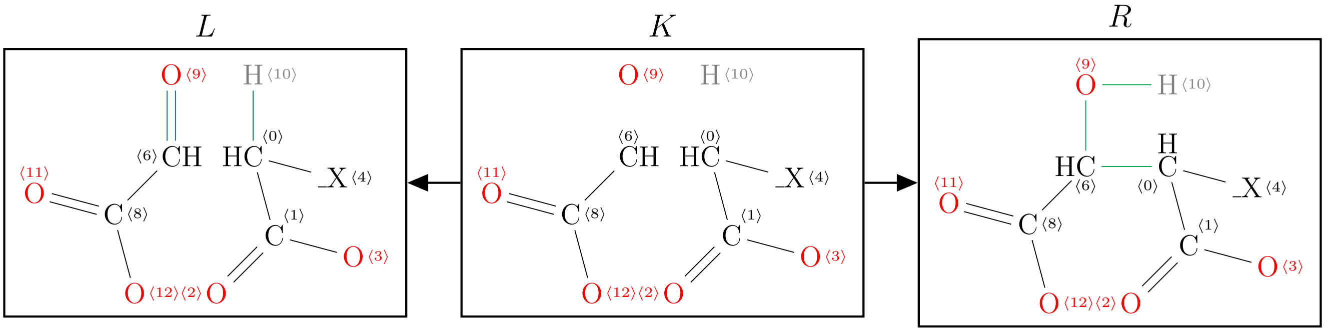

Files: out/112_r_54_11300110_{L, K, R}

_X ∈ {'H', 'C'}

0.1.39 GLYOX_alanine_2,3_aminomutase_01

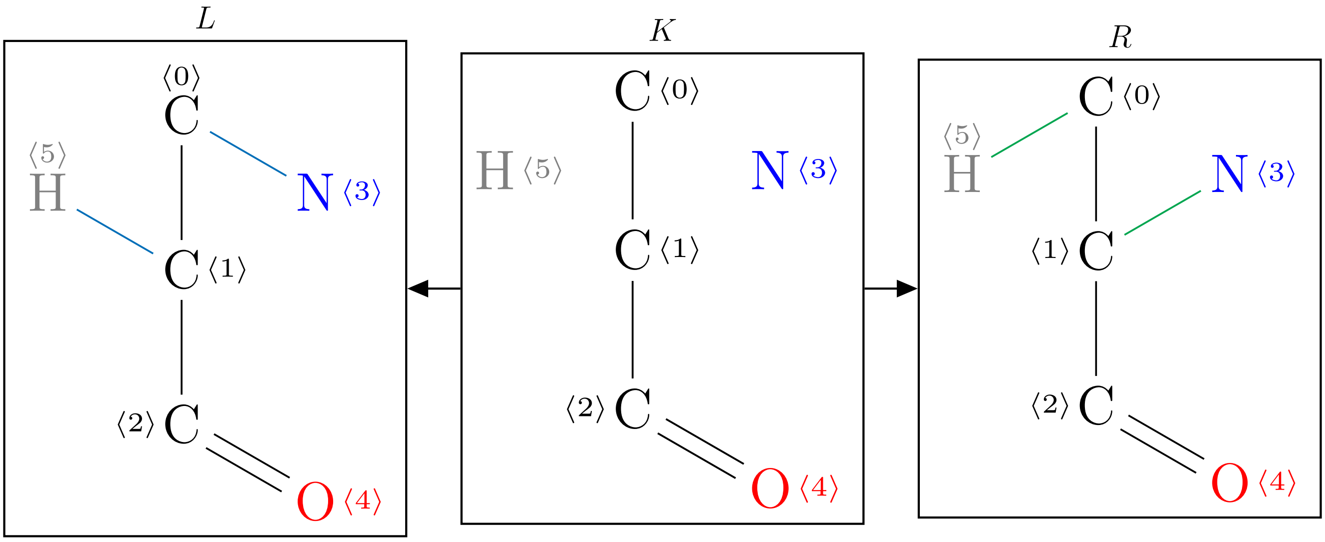

Files: out/115_r_55_11300110_{L, K, R}

0.1.40 GLYOX_transaminase_01

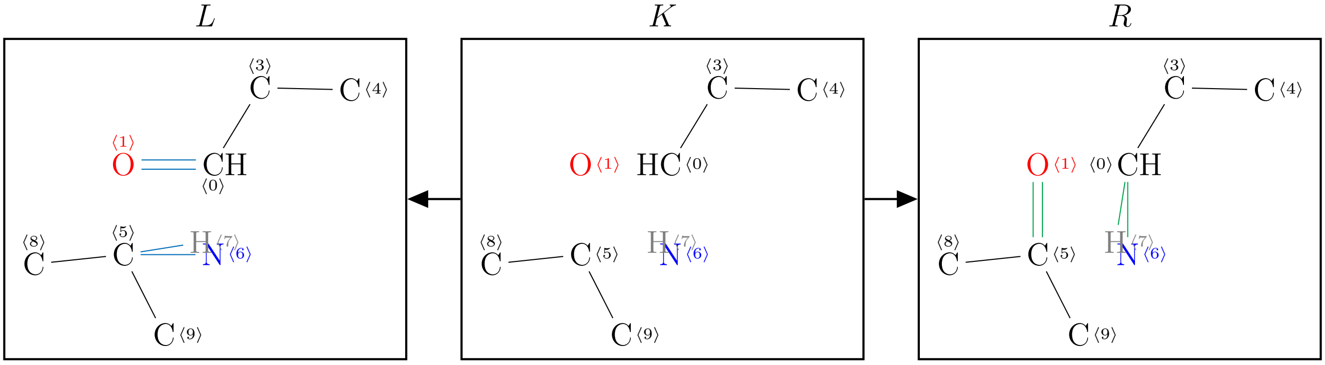

Files: out/118_r_56_11300110_{L, K, R}

0.1.41 GLYOX_transaminase_01

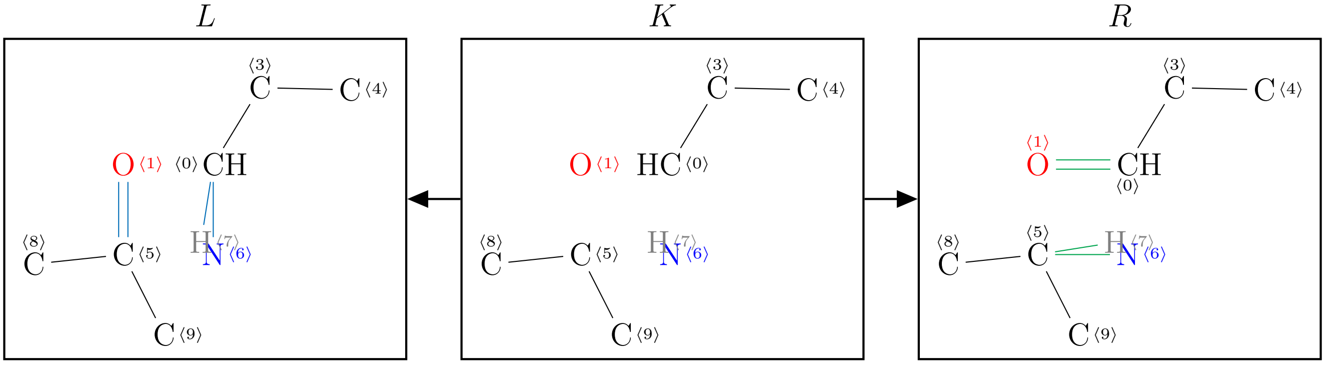

Files: out/121_r_57_11300110_{L, K, R}

0.1.42 GLYOX_methylmalonyl-CoA_carboxytransferase_01

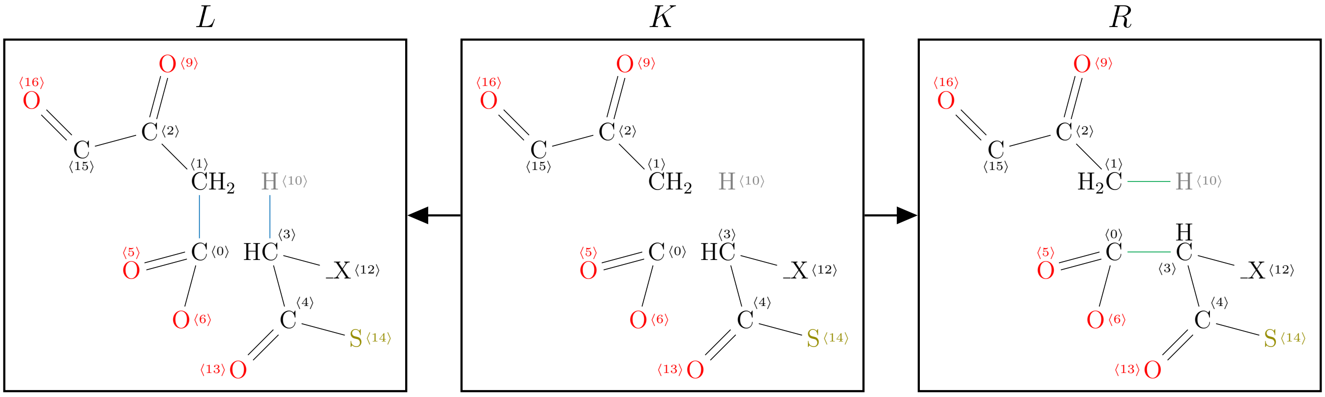

Files: out/124_r_58_11300110_{L, K, R}

`_X` ∈ {'H', 'C'}
